# Gavage of D-Ribose induces Aβ-like deposits, Tau hyperphosphorylation as well as memory loss and anxiety-like behavior in mice

**DOI:** 10.18632/oncotarget.6021

**Published:** 2015-10-07

**Authors:** Beibei Wu, Yan Wei, Yujing Wang, Tao Su, Lei Zhou, Ying Liu, Rongqiao He

**Affiliations:** ^1^ State Key Laboratory of Brain and Cognitive Sciences, Institute of Biophysics, Chinese Academy of Sciences, Beijing, China; ^2^ Key Laboratory of Mental Health, Institute of Psychology, Chinese Academy of Sciences, Beijing, China; ^3^ Alzheimer's Disease Center, Beijing Institute for Brain Disorders, Capital Medical University, Beijing, China; ^4^ University of Chinese Academy of Sciences, Beijing, China; ^5^ Animal Experiment Center, Core Facility for Protein Research, Institute of Biophysics, Chinese Academy of Sciences, Beijing, China

**Keywords:** D-Ribose, memory impairment, Tau hyperphosphorylation, Aβ-like deposition, anxiety-like behavior, Gerotarget

## Abstract

In addition to D-Glucose, D-Ribose is also abnormally elevated in the urine of type 2 diabetic patients, establishing a positive correlation between the concentration of uric D-Ribose and the severity of diabetes. Intraperitoneal injection of D-Ribose causes memory loss and brain inflammation in mice. To simulate a chronic progression of age-related cognitive impairment, we orally administered D-Ribose by gavage at both a low and high dose to 8 week-old male C57BL/6J mice daily for a total of 6 months, followed by behavioral, histological and biochemical analysis. We found that long-term oral administration of D-Ribose impairs spatial learning and memory, accompanied by anxiety-like behavior. Tau was hyperphosphorylated at AT8, S396, S214 and T181 in the brain. Aβ-like deposition was also found in the hippocampus for the high dose group. D-Glucose-gavaged mice did not show significant memory loss and anxiety-like behavior under the same experimental conditions. These results demonstrate that a long-term oral administration of D-Ribose not only induces memory loss with anxiety-like behavior, but also elevates Aβ-like deposition and Tau hyperphosphorylation, presenting D-Ribose-gavaged mouse as a model for age-related cognitive impairment and diabetic encephalopathy.

## INTRODUCTION

Alzheimer's disease (AD), at times referred to as type 3 diabetes, is the most common form of dementia in elderly people [1]. AD is characterized by an imbalance of the cellular energetic metabolism, but the cause for most AD cases is still unknown and less than 2.5% of all cases have a genetic origin [2]. AD patients suffer from memory loss, and other symptoms such as anxiety and depression [3]. Studies show that 25-60% of AD outpatients display anxiety symptoms. The proportion of anxiety symptoms among both hospitalized AD patients and those in the community is higher than that of healthy elderly peers [4].

Memory loss in AD and the underlying pathomechanisms have been widely studied. Many animal models, in particular transgenic mouse models, have been established to study memory loss, such as amyloid- β (Aβ)-depositing APP-transgenic [5] and APP/PS1-transgenic mice [6], neurofibrillary tangle-forming P301L Tau transgenic mice [7], and the senescence accelerated mouse-prone 8 strain (SAMP8) [8]. When the Tau and Aβ pathology are combined in triple transgenic mice (3xTg-AD), they display memory loss, anxiety-like behavior and subdued social behavior [9]. The Tg-APP (Sw, V717F)/B6 mouse also displays memory loss, anxiety-like behavior and decreased motor coordination [10].

Amyloid plaques and neurofibrillary tangles are key features of the AD brain [11]. Plaques consist of insoluble extracellular deposits of the Aβ peptide (Aβ) [12]. Tangles contain aggregated forms of the microtubule-associated Tau, which is hyperphosphorylated and accumulates intracellularly [13, 14]. A lot of studies have been carried out to search for risk factors in the production and aggregation of Aβ and/or phosphorylated Tau (pTau) [15]. Mice do not develop senile plaques with Aβ deposition even as they age, nor do they develop neurofibrillary tangles [16]. They only develop plaques or tangles when transgenic approaches are pursued as discussed above. Here, we asked ourselves whether it would be possible to nonetheless establish such a model simply by oral administration of D-Ribose.

Since glycation has been found to be involved in neurodegenerative diseases for instance AD, increased attention has been paid to the role of glycation in AD pathogenesis [17, 18]. The role is addressed of D-Ribose in glycation because it has higher activity in glycating proteins than D-Glucose [19, 20]. *In vitro* studies showed that D-Ribose induces protein misfolding rapidly leading to globular-like aggregations that are cytotoxic to neuronal cells [21]. Intraperitoneal injection of D-Ribose for 30 days revealed high levels of glycated proteins and advanced glycation end products (AGEs) in the blood and brain of wildtype mice. The mice also exhibited impairment of spatial learning and memory [22]. More recently, D-Ribose was found to be increased in the urine of type 2 diabetic patients [23], suggesting that diabetic patients may be suffered from metabolic imbalance of not only D-Glucose but also D-Ribose [24]. Therefore, we rationalized that the role of D-Ribose in glycation and diabetic complications, for instance encephalopathy [25], should be investigated.

D-Ribose is a key component of many important biomolecules including RNA, ATP and Riboflavin [26-28]. Many foods, such as wheat bran, eggs, meat, cheese and yeast, contain reasonable high concentrations of RNA and riboflavin. The pentose phosphate pathway can convert hexose to D-Ribose. Besides food, D-Ribose is orally administered to improve athletic performance and the ability to exercise by boosting muscle energy as a readily available source of energy. It is also used to improve symptoms of diseases such as chronic fatigue syndrome, fibromyalgia and coronary artery disease [29, 30]. To mimic the effects the intake of D-Ribose has in humans, complementary studies should be carried out in animals.

In the current study, we observed that long-term (6 months) gavage of D-Ribose at two different concentrations caused memory loss and anxiety-related behaviors, accompanied by Aβ-like deposition and Tau hyperphosphorylation in the hippocampus and cortex. Therefore, the accumulation of D-Ribose and its metabolic imbalance should be considered in the study of diabetic encephalopathy and age-related cognitive impairment or when a high dose of D-Ribose is used for long term energy supply.

## RESULTS

### No significant differences in body weights and motor abilities between experimental group and normal control

Based on Han and colleagues [22], D-Ribose administration through intraperitoneal injection impairs spatial learning and memory in mice. We wondered whether oral administration of D-Ribose would also affect cognitive function. First, C57BL/6J wildtype mice were gavaged with D-Ribose (low dose 0.375 g/kg·d and high dose 3.75 g/kg·d), D-Glucose (low dose 0.45 g/kg·d and high dose 4.5 g/kg·d) or saline as control for 6 months. None of the treatment groups showed any overt visual abnormalities. There was no difference in weight gain between the groups (Supplementary Table 1, *n* = 12, *P* > 0.05).

Second, to exclude the interference of motor abilities on the behavioral tests, we measured the muscle strength in the sugar-gavaged groups compared with the normal control group. No significant difference (*n* = 12, *P* > 0.05) was observed in the forelimb grip strength (Supplementary Figure 1a).

Third, to examine motor coordination, mice were allowed to perform the Rotarod test. D-Ribose-gavaged, D-Glucose-gavaged and normal mice showed similar latencies (Supplementary Figure 1b, *n*=12, *P* > 0.05). This demonstrates that D-Ribose- and D-Glucose-treated mice do not display any abnormal motor coordination and muscle strength compared to untreated mice.

### Oral administration of D-Ribose declining spatial learning and memory

**Figure 1:**
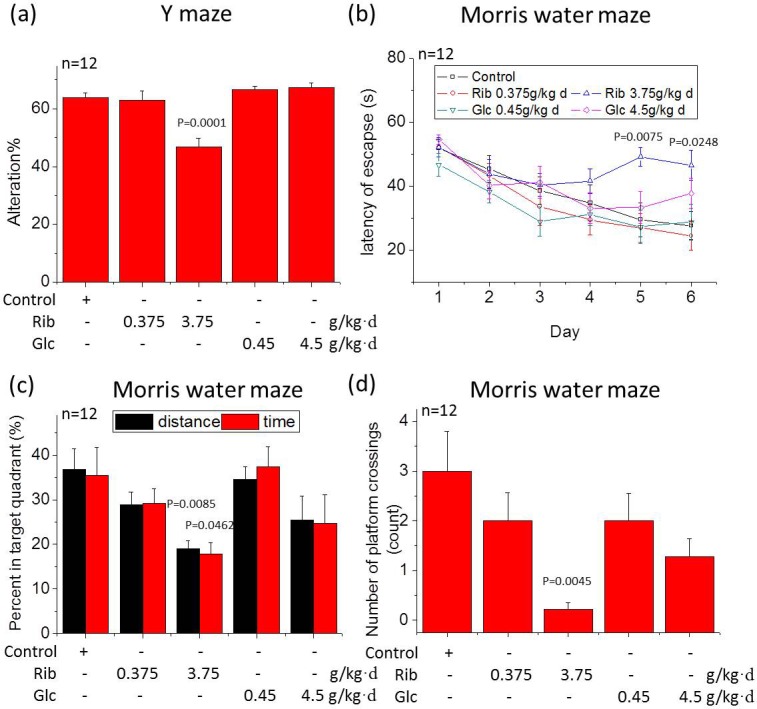
Reduced spontaneous alteration in the Y maze test and impaired spatial memory in Morris water maze in mice gavaged with D-Ribose Mice were gavaged for 6 months with two concentrations of D-Ribose (0.375 and 3.75 g/kg·d) and D-Glucose (0.45 and 4.5 g/kg·d), using saline as control. The percentage of alteration in the Y maze was used to measure the exploration of a new environment (a). The length of time needed to find the hidden platform was recorded as escape latency for each of the six training days (b). The percentage of search time and the distance spent in the quadrant from which the platform was removed during the probe trial is shown (c), as well as the number of platform crossing in the probe trail is shown (d). All values are expressed as mean±S.E.M. The P values are obtained from comparative analysis (two-way ANOVA with post' hoc test) of indicated group with the controls.

To determine whether oral administration of D-Ribose affects learning and memory of mice, the Y maze spontaneous alteration test as described [31] and the Morris water maze were performed (Figure 1). D-Ribose-treated mice (3.75 g/kg·d) showed significantly less alteration compared with other experimental groups and normal control (Figure 1a, *n* = 12, *P* = 0.0001). Total arm entries did not differ (data not shown). According to Stewart and colleagues [32], a high dose of D-Ribose administration declines memory functions in mice.

We next used a more robust test of spatial memory, the Morris water maze. Before the test, we confirmed that there was no overt phenotypical difference between the groups that would indicate a clinical impairment (*n* = 12, *P* > 0.05). During the training sessions, all mice improved their performance as indicated by increasingly shorter escape latencies over successive days. Mice from each treatment group had similar levels of performance (no significant individual effect was observed in the first three trials on day 1, *n* = 12, *P* > 0.05) prior to the test. The escape latency of mice treated with D-Ribose (3.75 g/kg·d) on day 5 and 6 was higher than that of the control group (*n* = 12, *P* = 0.0075, *P* = 0.0248). However, there was no significant difference between the control group and the three groups employed (Figure 1b, *n* = 12, *P* > 0.05).

Withdrawal of the platform induced a general tendency to swim in the quadrant where the platform was previously located and in the platform zone. D-Ribose (3.75 g/kg·d)-treated mice spent significantly less time (*n* = 12, *P* = 0.0462) and distance (*n* = 12, *P* = 0.0085) swimming in the target quadrant than controls, D-Ribose (0.375 g/kg·d)- and D-Glucose (0.45 and 4.5 g/kg·d)-treated mice (Figure 1c). The number of platform crossings was also lower in the D-Ribose (3.75 g/kg·d)-treated group compared with the other groups (Figure 1d, *n* = 12, *P* = 0.0045). These results indicate that learning and memory in D-Ribose-treated mice are impaired.

### D-Ribose-gavaged mice showing anxiety-like behaviors

**Figure 2:**
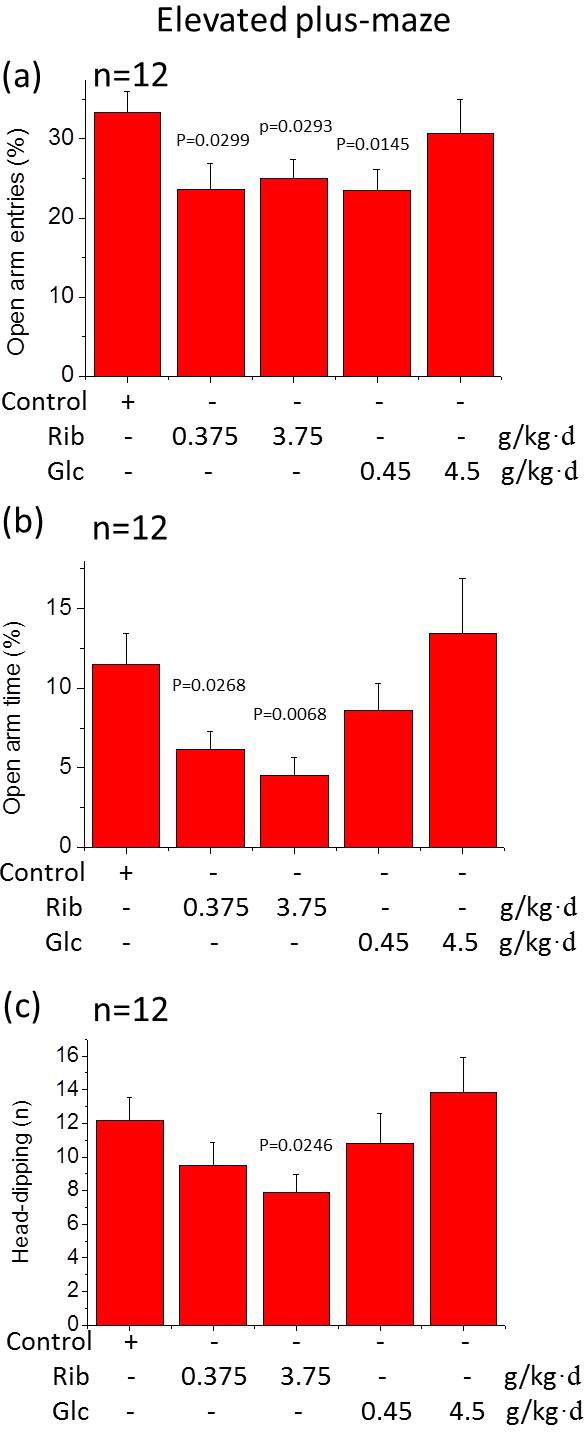
Elevated plus-maze test Mice were gavaged with D-Ribose or D-Glucose for 6 months as described in Figure 1. In the elevated plus-maze, the percentage of the time spent (min) in the open arms (a), the number of open arm entries (b) and head dipping (c) within 5 min was recorded. All values are expressed as mean±S.E.M. The P values are obtained from comparative analysis of indicated group with the controls.

Anxiety commonly accompanies memory loss in AD patients. To investigate whether D-Ribose administration leads to anxiety-related behavior, the mice were tested in the elevated plus-maze. D-Ribose (0.375 g/kg·d and 3.75 g/kg·d)-treated mice showed lower numbers of entries into the open arms (*n* = 12, *P* = 0.0299, *P* = 0.0293). The D-Glucose (0.45 g/kg·d)-treated group also showed a decreased number of entries (*n* = 12, *P* = 0.0145) (Figure 2a). To determine whether both D-Ribose- and D-Glucose-treated mice suffer from anxiety, the time of the mice spent in the open arms was also measured (Figure 2b). A significant reduction in time spent in the open arms was found for D-Ribose (0.375 g/kg·d and 3.75 g/kg·d)-treated mice (*n* = 12, *P* = 0.0268, *P* = 0.0068), but not the D-Glucose-treated mice. Furthermore, the number of head-dipping below the maze was significantly reduced in the D-Ribose (3.75 g/kg·d)-treated mice (*n* = 12, *P* = 0.0246) (Figure 2c). No effect was observed in all other groups (*P* > 0.05).

To assess whether D-Glucose-treated mice also display an anxiety-related behavior, an open field arena test was performed, in which the decrease in the time spent in the central area of the arena is considered as an index of anxiety. Mice with D-Ribose (0.375 g/kg·d)-administration spent significantly less time in the central zone, and travelled less distances within the center of the arena (n = 12, *P* = 0.0022, *P* = 0.0039) (Supplementary Figure 2a, b). However, mice treated with both low and high dose D-Glucose did not show any alterations in this test (*n* = 12, *P* > 0.05). To determine whether D-Ribose affects the behavior in the open field test, we counted the entries of the square centre. Both low and high doses of D-Ribose significantly decreased the square centre entries in mice (*n* = 12, *P* = 0.0001, *P* = 0.0004), but the entries were not D-Ribose concentration dependent (Supplementary Figure 2c).

### Neither D-Ribose- nor D-Glucose-gavaged mice displaying depression-like behavior

Depression has long been known to affect learning and memory [33]. To clarify whether the learning and memory results from depression or not, we performed the forced swim test [34]. The time spent immobile was increased in the D-Ribose (3.75 g/kg·d)-administrated mice compared with controls suggesting that D-Ribose might induce depression-like behavior (*n* = 12, *P* = 0.022) (Supplementary Figure 3). To assess this further, we carried out the tail suspension test, referring to the duration of immobility as ‘despair’. The analysis showed that D-Ribose treatment did not cause a marked increase in the immobility time compared to D-Glucose-treated groups and control (*n* = 12, *P* > 0.05) (Supplementary Figure 4a). None of the mice showed significant differences in the power of movements (*n* = 12, *P* > 0.05) (Supplementary Figure 4b). Taking all these findings together, it cannot be concluded that oral administration of D-Ribose induces a depression-like behavior in mice.

### D-Ribose treatment inducing Tau hyperphosphorylation in hippocampus and cortex

The presence of hyperphosphoylated Tau and the concomitant formation of neurofibrillary tangles are closely related to cognitive impairments. We therefore determined Tau phosphorylation in brain by Western blotting analyses.

**Figure 3:**
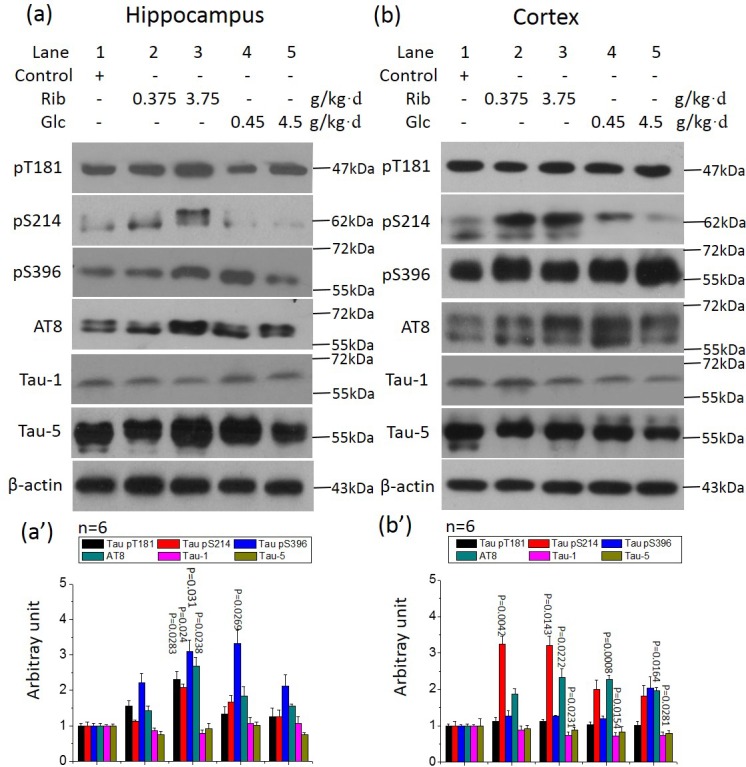
Tau phosphorylation in the hippocampus of D-Ribose- and D-Glucose-treated mice Conditions for the treatment were the same as for Figure 1, except that phosphorylation of Tau in the hippocampus and cortex was detected by Western blotting using anti-pT181, anti-pS214, anti-pS396, anti-AT8, anti-Tau-1 and anti-Tau-5 (a, b), respectively. β-Actin was used as a loading control. Quantification is shown in a' and b'. The saline control value was set as 1.0. The phosphorylation levels were expressed as the ratio between phospho-site and the total Tau staining. A full picture of the blots can be found in the Supplementary Information. All values are expressed as mean±S.E.M. The P values are obtained from comparative analysis of indicated group with the controls.

First, using the monoclonal antibody Tau-5, we found no significant difference in total Tau levels between the experimental and control groups (*P* > 0.05) (Figure 3a, 3b). Full-length blots were presented in Supplementary Figure 14 and 15. Next, we used the monoclonal antibody Tau-1 (recognizing amino acid residues 189-207) that detects dephosphorylated forms of Tau. The signals of Tau-1 were decreased in the cortex of both the D-Ribose (3.75 g/kg·d)- and D-Glucose-treated groups (*n* = 6, *P* = 0.0231, *P* = 0.0154, *P* = 0.0281) (Figure 3b), and slightly decreased in the hippocampus of D-Ribose (3.75 g/kg·d)-treated mice (*n* = 6, *P* > 0.08) (Figure 3a). This indicates that cellular Tau protein should be more phosphorylated in these groups.

**Figure 4:**
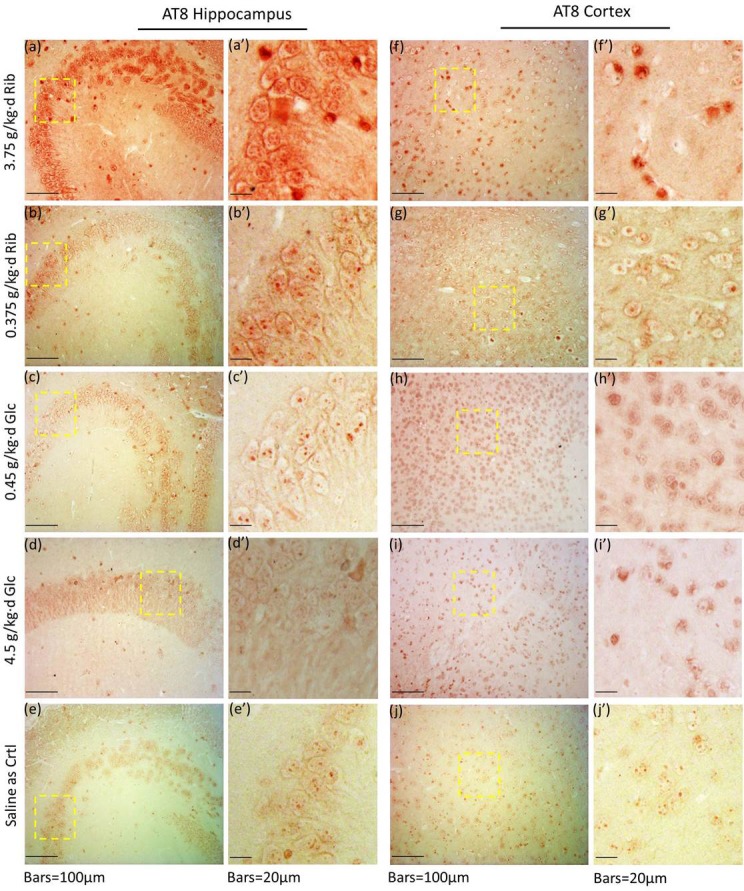
Immunohistochemical staining of AT8 in hippocampus and cortex Mice were gavaged with D-Ribose at 3.75 g/kg·d (a, a', f, f'), D-Ribose at 0.375 g/kg·d (b, b', g, g'), D-Glucose at 0.45 g/kg·d (c, c', h, h'), D-Glucose at 4.5 g/kg·d (d, d', i, i') and saline (e, e', j, j') for 6 months, and the hippocampal and cortex slices were prepared as described in Materials and Methods. AT8 in the hippocampus (a, a'-e, e') and cortex (f, f'-j, j') was detected by immunohistochemistry using anti-Tau [AT8] polyclone antibody.

Therefore, in order to investigate whether Tau was hyperphosphorylated, we used antibody AT8 to detect Tau phosphorylated at epitopes Ser199 and Ser202, the sites that represent one of the earliest neuropathological changes, with a pivotal role in the initial pathogenesis of AD [35]. AT8 levels were significantly increased in the hippocampus and cortex of D-Ribose (3.75 g/kg·d)-treated mice (*n* = 6, *P* = 0.0238, *P* = 0.0222) (Figure 3). AT8 levels were also increased in the cortex of the two D-Glucose (0.45 g/kg·d and 4.5 g/kg·d)-treated groups, but not in their hippocampi (Figure 3). To further analyze the role D-Ribose has in AT8 phosphorylation, we performed the immunohistochemical staining of brain sections. AT8 immunoreactivity (in red) was evident in hippocampal and cortical neurons after the mice had been treated with D-Ribose (3.75 g/kg·d) for 6 months (Figure 4a, 4a', 4f, 4f'). A slight increase in AT8 reactivity was also observed in the hippocampi and cortices of mice treated with the lower dose of D-Ribose (0.375 g/kg·d) (Figure 4b, 4b', 4g, 4g'). However, AT8-immunoreactivity was not strong in the cortex and hippocampus of D-Glucose-treated mice (Figure 4c, 4c', 4h, 4h', 4d, 4d', 4i, 4i'), and it was almost absent in controls (Figure 4e, 4e', 4j, 4j').

We also explored the phosphorylation of Ser396 (pS396), an epitope with a key role in the formation of paired helical filaments in AD [36]. Levels of pS396 were significantly (*n* = 6, *P* = 0.031, *P* = 0.0269) increased in the hippocampi of D-Ribose (3.75 g/kg·d)- and D-Glucose (0.45 g/kg·d)-treated mice (Figure 3a), but no increase was found in the cortex (*n* = 6, *P* > 0.05). By immunohistochemistry, with β-actin staining as negative control (Supplementary Figure 5), except for the intracellular accumulation of pS396 in hippocampi (and to some degree in cortex) of D-Ribose (3.75 g/kg·d)- treated mice (Supplementary Figure 6a, 6a', 6f, 6f'), there was no increased phosphorylation in the other experimental mice compared to controls (Supplementary Figure 6b, 6b'-6e, 6e'; 6g, 6g'-6j, 6j').

As a third epitope, we assessed phosphorylation of Ser214 (pS214) that is unique to AD and not found in normal Tau [37]. Levels of pS214 were significantly increased in the hippocampi (3.75 g/kg·d) and cortex of D-Ribose (3.75 g/kg·d and 0.375 g/kg·d)-treated mice (*n* = 6, *P* = 0.024, *P* = 0.0042, *P* = 0.00143) (Figure 3a, b). We also employed immunohistochemistry and found that, except for the intracellular accumulation of pS214 in hippocampi of D-Ribose (3.75 g/kg·d and 0.375 g/kg·d)-treated mice (Supplementary Figure 7a, 7a', 7b, 7b'), where there were no overt changes observed in the other experimental groups compared to controls (Supplementary Figure 7c, 7c'-7e, 7e'; 7f, 7f'-7j, 7j').

Finally, we determined levels of phospho-Thr181 (pT181), which is believed to be a reliable marker in predicting the conversion from mild cognitive dysfunction to AD [38]. Levels of pT181 were significantly increased in the hippocampi of D-Ribose (3.75 g/kg·d)-treated mice (*n* = 6, *P* = 0.0283) (Figure 3a). The immunohistochemical analyses revealed an increase of immunoreactivity for pT181 in hippocampi of D-Ribose (3.75 g/kg)-treated mice (Supplementary Figure 8a, 8a'). However, the hippocampal (Supplementary Figure 8b, b'-d, d') and cortical (Supplementary Figure 8f, 8f'-8i, 8i') sections of the other experimental groups did not show increased immunoreactivity compared to the controls (Supplementary Figure 8e, 8e'; 8j, 8j').

The data collectively show that oral administration of high dose D-Ribose triggers the hyperphosphorylation of Tau in mice. Under the tested conditions, the oral administration of D-Ribose to mice is much more effective in triggering Tau hyperphosphorylation than D-Glucose, especially in the hippocampus.

### Amyloid-β-like products elevation in hippocampus in the presence of D-Ribose

**Figure 5:**
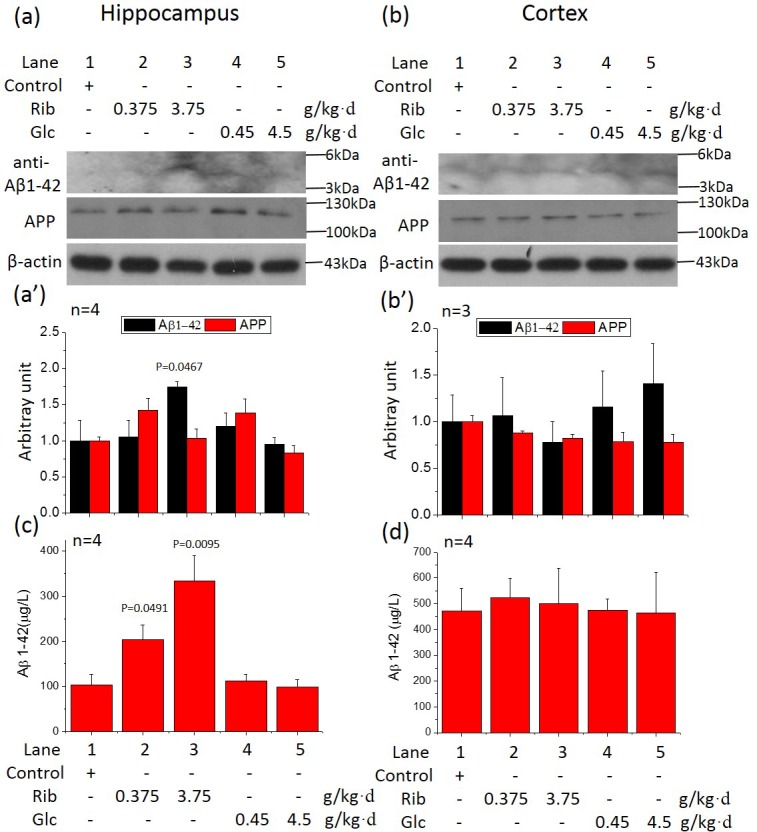
Changes in amyloid-β (Aβ) like product levels in the brain of sugar-treated mice Conditions for the treatment were the same as those given in Figure 1, except that the expression of Aβ1-42 and APP in hippocampus (a) and cortex (b) were detected by Western blotting using anti-Aβ_1-42_ polyclonal antibody and anti-APP polyclonal antibody. β-Actin was used as a loading control. Quantification is shown in a' and b'. The saline control value was set as 1.0. A full picture of the blots can be found in the Supplementary Information. Aβ-like product levels were assayed in hippocampus (c) and cortex (d) by ELISA. All values are expressed as mean±S.E.M. The P values are obtained from comparative analysis of indicated group with the controls.

**Figure 6:**
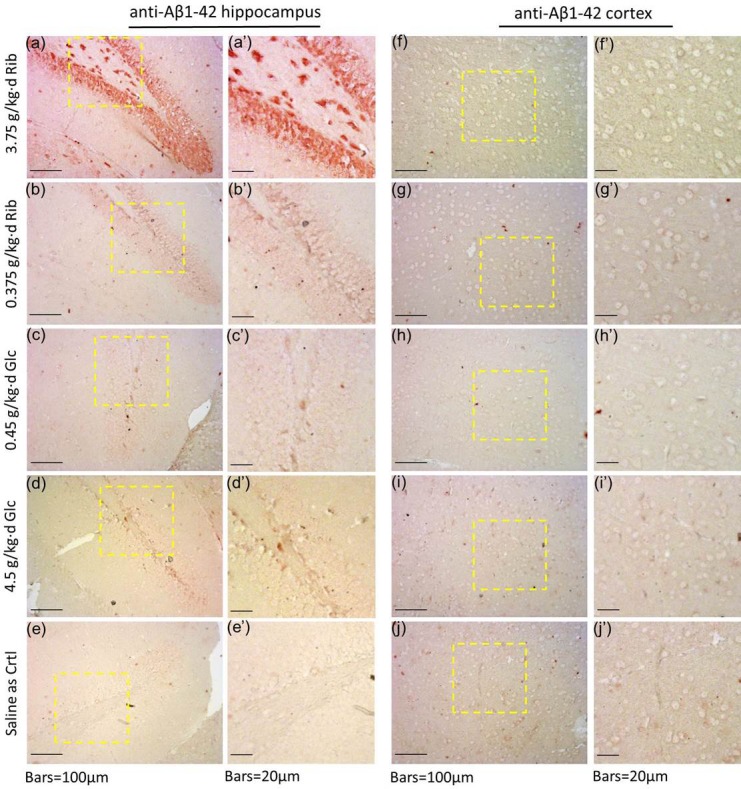
Immunohistochemical staining of Aβ-like deposits in hippocampus and cortex Conditions for the treatment were the same as those given in Figure 4. Aβ-like deposits in the hippocampus (a, a'-e, e') and cortex (f, f'-j, j') were detected by immunohistochemistry using anti-Aβ1-42 polyclonal antibody.

Amyloid-β is one of the major factors in the progression of AD pathology. To clarify whether D-Ribose induces the accumulation of Aβ besides Tau hyperphosphorylation, we measured the Aβ content in the mouse brain with the antibody against Aβ1-42 after administration of sugar. Aβ levels were significantly elevated in the hippocampus of D-Ribose (3.75 g/kg·d)-treated mice compared with controls (*n* = 6, *P* = 0.0317) (Figure 5a, Supplementary Figure 9). However, APP levels were not markedly increased (*n* = 6, *P* = 0.906). In the cortex, neither Aβ nor APP levels were significantly increased in both D-Ribose- and D-Glucose-gavaged mice (Figure 5b). Full-length blots were presented in Supplementary Figure 16. To further demonstrate the elevated Aβ-like product levels in the D-Ribose-gavaged mice, the ELISA was used. Aβ-like product levels were also significantly elevated in the hippocampus of D-Ribose (both 0.375g/kg·d and 3.75 g/kg·d)-treated mice compared with controls (*n* = 4, *P* = 0.0491, *P* = 0.0095) (Figure 5c). In cortex, Aβ-like product levels were not markedly increased (Figure 5d). The selective elevation of Aβ-like product in the hippocampus may contribute to the D-Ribose-mediated impairment in learning and memory. In the hippocampus, we observed immunoreactivity for Aβ-like product in the D-Ribose (3.75 g/kg·d)-treated group, different from all other treatment groups (Figure 6). This demonstrates that administration of D-Ribose can promote Aβ-like deposition in the hippocampus but not the cortex.

### No significant differences in blood D-Glucose and serum AGEs levels between D-Ribose- and D-Glucose-gavaged mice

We measured and recorded concentrations of blood D-Glucose in mice (normal fasting blood sugar levels of 5~10 mmol/L) during the 6-month administration (Supplementary Table 2). No significant difference in the concentration of blood sugar was observed between the D-Ribose- and D-Glucose-gavaged groups (*n* = 12, *P* > 0.05) (Supplementary Figure 10a, b). The changes in fasting blood sugar were in the normal range (5~10 mmol/L) even though significant decreases were observed in all sugar treatment groups from the second month compared with control. This indicates that administration of either D-Ribose or D-Glucose did not induce hyper- or hypo-glycemia under our experimental conditions. We also measured the concentration of serum insulin (Supplementary Figure 11). Only D-Ribose (3.75 g/kg·d)-treated mice showed a slight increase in serum insulin compared with controls that were however not significant. No significant difference in fasting blood sugar levels was found between D-Ribose (3.75 g/kg·d)- and D-Glucose (4.5 g/kg.d)-gavaged mice, suggesting that the slight increase in insulin levels did not markedly affect levels of blood sugar.

To further investigate whether oral administration of D-Ribose enhances the glycation of serum proteins, we measured levels of AGEs in different mouse tissues. Levels of AGEs in the serum did not differ between the groups. Marked changes in AGEs were not found in cortex, hippocampus and kidney either, except for liver where AGEs increased in the 4.5 g/kg·d D-Glucose group (Supplementary Figure 12). These results indicate that oral administration of D-Ribose does not induce overweight, hyper- or hypo-glycemia and AGE accumulation, compared to D-Glucose and the control under our experimental conditions. AGEs might not be involved in memory impairment and anxiety-like behavior of mice that have been administered D-Ribose orally.

## DISCUSSION

In the current study, we showed that feeding D-Ribose daily through gavage to mice for 6 months is correlated with cognitive impairment. Mice treated with D-Ribose (3.75 g/kg·d) exhibited learning and memory decline and anxiety-like behavior, accompanied by Aβ-like deposition and Tau hyperphosphorylation in their brain, especially the hippocampus. These features are AD-like, both with regards to pathology (abnormal modifications and aggregations of Tau protein and Aβ peptide) and pathophysiology (memory loss accompanied by anxiety-like behavior). This suggests that D-Ribose-gavaged mouse model may be useful to study age-related cognitive impairment [39] and diabetic encephalopathy, conditions in which Aβ deposition and Tau hyperphosphorylation are found [40, 41].

The D-Ribose-induced high level of Aβ-like deposition in the hippocampus demonstrates that mice can develop Aβ-like deposits even though the APP gene sequence of mice differs from that of humans [16, 42]. Previously, we employed mice in a methanol-gavage experiment and found that methanol can distinctly promote hyperphosphorylation of Tau, but not Aβ deposition [43]. However, by oral administration of D-Ribose, Aβ-like deposition can be induced under the experimental conditions. Amyloid β deposit is clear and robust (Figure 6). The anti-Aβ antibody employed may recognize Aβ monomer and the polymers in immunohistochemical sections, since there are much higher molecular masses of polymers besides a slight band of 4kDa Aβ (Supplementary Figure 16).

D-Ribose is significantly increased in the brain of D-Ribose-treated mice (Supplementary Figure 13). However, it did not markedly increase AGE levels in the brain, serum, liver and kidney under the experimental conditions. However, intraperitoneal injection of similar concentrations of D-Ribose for 30 days, as described [22], resulted in AGE accumulation in the mouse blood and brain. Whether D-Ribose elevates levels of AGEs in mice or not depends on the mode of administration. Thus, impairment of cognitive ability by oral administration of D-Ribose is probably not related to brain AGE accumulation, but rather the abnormally high levels of Aβ-like deposition and Tau hyperphosphorylation in mouse brain (Supplementary Paragraph 1).

To compare the effects between D-Ribose and D-Glucose, oral administration of D-Glucose did not affect cognition. Even though D-Glucose showed elevated pS396 levels in the hippocampus and AT8 reactivity in the cortex, the elevations were not clearly seen by immunohistochemical staining of sections using antibodies AT8, pS396, p214 and T181 (Figure 4, Supplementary Figure 6-8). D-Glucose was found to reduce the percentage of open arm entries of mice in the elevated plus-maze test (Figure 2). However, the D-Glucose-gavaged mice did not show an abnormal behavior in the open field test (Supplementary Figure 2), indicating that D-Glucose-gavaged mice may not be anxious. Oral administration of D-Glucose did not cause high levels of Aβ-like deposits. In contrast, gavage of D-Ribose resulted not only in high levels of Aβ-like deposition and Tau phosphorylation in the hippocampus, but also in memory loss and anxiety-like behavior.

The long-term administration of D-Ribose did not cause any significant changes in the body weight compared to mice treated with D-Glucose. Fasting blood sugar levels and AGE levels did not differ between the groups. Except for memory loss and anxiety, both D-Ribose- and D-Glucose-gavaged mice did not show additional abnormal behavior such as motor dysfunction, reduced muscle strength and impaired co-ordination. Even though D-Glucose-gavaged mice showed a decrease in the open arm entries (Figure 2b), they behaved normally in the open field test. Thus, D-Glucose-induced depression-like behavior is not supported by our study.

As shown in Supplementary Figure 11, the blood insulin level was slightly increased in D-Ribose-treated mice (3.75 g/kg·d). As described by Segal and colleagues [44], D-Ribose can cause insulin release and decrease blood D-Glucose. Our results are similar to their findings (Supplementary Table 2). Since mice were gavaged with the sugar, it needs more insulin to regulate it. The secreted insulin induced lower level of blood sugar [44, 45].

In AD, memory disorder is known as the most important symptom. Behavioral and psychiatric symptoms like anxiety and depression may also follow the memory loss. In this study, D-Ribose-treated mice exhibit an anxious symptom in open field test and elevated plus maze. Open field test on D-Ribose-treated mice was equivocal though counts of center square entries gave some positive results. Elevated plus maze shows the data in a D-Ribose concentration dependent manner. However, the data obtained with these tests were often contradictory as described by Carola and colleagues [46]. For our data, it is possible that the elevated plus-maze test was more suitable to exhibit the anxiety-like behaviors for D-Ribose-gavaged mice. Of course, the anxiety-like effect of D-Ribose on mice should be further investigated. Forced swim test showed that the mice under D-Ribose treatment behave more immobile, which indicates the depression state. While tail suspension test did not show any difference in the immobility time. Administration of D-Glucose as a control did not show anxiety-like behavior and significant depression state. Take all those results, oral administration of D-Ribose induces not only memory impairment but also anxious behavior in mice.

Diabetes is a group of metabolic diseases in which there are high blood sugar levels over a prolonged period. Diabetic encephalopathy is one of the serious diabetic complications leading to memory loss [32] and anxiety [47]. Tau hyperphosphorylation and Aβ deposition in the diabetic brain have been reported [40, 41, 48]. Recently, we found that type 2 diabetic patients have abnormally high levels of uric Ribose [49] which is positively correlated with the severity of diabetes [24]. Oral administration of D-Ribose to mice also causes impaired memory functions, Tau hyperphosphorylation and Aβ-like deposition. These data suggest that an imbalance of D-Ribose metabolism may play a role in diabetic encephalopathy.

In summary, oral administration of D-Ribose leads to the impairment of cognitive ability and anxious behavior in mice. Meanwhile, D-Ribose-gavaged mice suffer from both Aβ-like deposition and Tau hyperphosphorylation in their brain, especially in the hippocampus. These abnormal modifications and the pathological aggregation of these proteins are probably correlated with the memory loss and anxiety-like behavior after D-Ribose treatment. Oral administration of D-Ribose to mice or rats is conveniently performed with a highly reproducible phenotype which can be used as an animal model for cognitive impairment and diabetic encephalopathy (Supplementary Paragraph 2).

## MATERIALS AND METHODS

### Ethics statement

The handing of mice and experimental procedures have been approved by the Animal Welfare and Research Ethics Committee of the Institute of Biophysics, Chinese Academy of Sciences (Permit Number: SYXK2013-77), and the methods were carried out in accordance with the approved guidelines.

### Animals and administration of D-Ribose and D-Glucose

Male C57BL/6J mice (8 weeks) were obtained from Vital River Laboratory Animal Technology Co. Ltd. (Beijing, China). The mice were randomly divided into five groups, and spent four days to acclimatize to the cages afterwards. Then, the mice received a daily gavage administration for 6 months of D-Ribose (0.375 g/kg or 3.75 g/kg), D-Glucose (0.45 g/kg or 4.5 g/kg), or saline (as control). All mice were maintained under pathogen-free conditions. We have described this procedure in detail in Supplementary Methods.

### Measurement of body weight and blood sugar/insulin concentrations

Body weight and blood sugar concentrations were recorded once per month from 8 to 32 weeks of age for mice. Fasting blood D-Glucose of mice were tested using a Sannuo safe blood D-Glucose meter (Changsha Sannuo Biological Sensing Technology Co., LTD, China), and the blood insulin concentration was detected by radioimmunoassay according to the instruction of Fred Clinical Inspection Institution (Hebei, China).

### Measuring grip strength

A tension meter (Bioseb, France) was used to test the forelimb grip strength in mice as described by Tilson and colleagues [50]. Mice were held at their tails and their front paws grasped the grid. Five grip force measurements were made and the LCD screen of the tension meter automatically displayed the maximum tensile strength at each time. The average of five measurements was taken to represent the forelimb grip strength.

### Rotarod test

As described by Guo and colleagues [34], mice were trained for 2 days on the Rotarod (Panlab, Spain) at a fixed speed of 10 r.p.m. for 5 min three times a day. On the third day, the mice were tested on the Rrotarod at a speed accelerating from 4 to 40 r.p.m. over the course of 5 min. The latency for each mouse to fall from the rod was recorded.

### Y-maze

The Y-maze we used was composed of three equally spaced arms (120°^−^; 40 cm long × 5 cm wide × 15 cm high). Activity in a Y-maze (Huaibeizhenghua biological instrument equipment Co., LTD, China) was used to measure spontaneous alternation performance (spatial working memory) and locomotor activity. The mice were placed in one of the arm compartments and allowed to move freely for 6 min. The sequence of arm entries was manually recorded. Alternation was defined as an entry into all three arms in consecutive choices. Spontaneous alternation percentage (SA%) was defined as the ratio of the arm entry choices that differed from the previous two choices to the total choices. The number of maximum spontaneous alternation was then calculated as the total number of arms entered minus 2 and the percentage was calculated as: actual alternations/maximum alternations×100 [51].

### Open field test

Mice were subjected to an open field test in an apparatus that consisted of a 50×50 cm open arena with 40 cm-high walls (Huaibeizhenghua biological instrument equipment Co., LTD, China). The entire test arena was adjusted to ensure even illumination. The mice were placed in the center of the arena, and their activity was recorded for 5 min. For video analysis, the open field arena was divided into 16 equal squares, and the centre square was defined as the central zone. Average velocity and total distance traveled were used as measures of overall motor activity. Activity in the central zone is usually regarded as a measure of anxiety.

### Elevated plus maze

The elevated plus maze (EPM) is made of four arms (30×6 cm, Huaibeizhenghua Biological Instrument Equipment Co., LTD, China) that are arranged in the configuration of a ‘+’ sign, comprising two open arms across from each other and perpendicular to two closed arms with a center platform (5×5 cm). The open arms had neither side nor end walls, whereas the closed arms had side and end walls (15 cm in height) but were open on the top. The mice were placed in the central square facing the corner between a closed and an open arm. Numbers of entries, which were defined as all four paws being positioned within one arm and the time spent on each arm, were measured. The number of rearing in the closed arms and of head-dipping was also measured at the same time [34].

### Morris water maze

The Morris water maze test was performed as described previously [52]. The apparatus consisted of a circular water tank (100 cm in diameter and 35 cm in height), containing water (23±1°C) to a depth of 15.5 cm, which was rendered opaque by adding white food dye. A platform (4.5 cm in diameter and 14.5 cm in height) was submerged 1 cm below the water surface and placed at the midpoint of one quadrant. Mice were exposed to a visual platform before they were exposed to a hidden platform. The visual platform was the same as in the hidden platform but a ‘flag’ was mounted that extends above the water surface by approximately 12 cm. Each mouse had four trials per day in the visual platform test for four consecutive days. In the hidden platform test, each mouse received four periods of training per day for six consecutive days. The latency to escape from the water maze (that is finding the submerged escape platform) was calculated for each trial. On day 7, a probe test was carried out by removing the platform and allowing each mouse to swim freely for 60 sec. The time that mice spent swimming in the target quadrant (where the platform had been located during the hidden platform training) was measured. All data were recorded with a computerized video system.

### Tail suspension test

The tail suspension apparatus consisted of a box of 20×20×30 cm and the test was performed as described [53]. The duration of immobility and the power of movement were recorded for 6 min using the Bioseb vision 3.0 system. Immobility was defined as the absence of any limb or body movements, except those caused by respiration. The power of movement was analysed in terms of force, energy and power developed over time, discriminating a behavior made up of weak and long lasting movements, from one of brief and intense movements.

### Forced swim test

The forced swim apparatus comprised of a clear plexiglas cylinder (25 cm in height, 10 cm in diameter). The mice were placed in this apparatus, which was filled with 24°C water to a depth of 15 cm. Fresh water was used for each mouse. In a 6-min test session, the first 2 min were designated as a habituation period and the duration of immobility during the final 4 min was recorded [34].

### Sample collection from animals

After the behavioral testing, the mice were sacrificed and their blood was collected as described previously [54] and centrifuged (4,000 r.p.m, 15 min, 20°C). Serum was aspirated and stored at −80°C until assayed. At the same time, the hippocampus, cortex, liver and kidney were quickly dissected either for subsequent Western blotting, or they were fixed in 4% paraformaldehyde for subsequent immunohistochemistry.

### Gel electrophoresis and Western blotting

Levels of AGEs in hippocampus, cortex, liver, kidney and serum were determined by Western blotting using standard protocols. The same method was used to analyze the expression of phosphorylated T181 (pT181), S214 (pS214), S396 (pS396), and Ser199/202 (AT8), or dephosphorylated Ser199/202 (Tau-1) (recognizing amino acid residues 189-207), Tau-5 (total Tau), APP and β-actin in hippocampus and cortex tissues of mice. Antibodies used were: mouse anti-AGE monoclonal antibody 6D12 (TransGenic, Japan) rabbit anti-pT181 polyclonal antibody (SAB, USA), rabbit anti-pS214 polyclonal antibody (Invitrogen, USA), rabbit anti-pS396 polyclonal antibody (Invitrogen, USA), rabbit anti-AT8 polyclonal antibody (Invitrogen, USA), mouse anti-Tau-1 monoclonal antibody (Millipore, USA), mouse anti-Tau-5 monoclonal antibody (Millipore, USA), rabbit anti- APP polyclonal antibody (CST, USA) and mouse anti-β-actin monoclonal antibody (Sigma, USA). We have described this procedure in detail in Supplementary Methods.

### Immunoblot detection of Aβ

Immunoblot detection of Aβ in brain tissues was as described [55]. Hippocampus and cortex samples were homogenized (150 mM NaCl with protease inhibitor cocktail in 50 mM Tris, pH of 8.0) and centrifuged (300,000*g* × 45 min), and the supernatant was removed. The pellet was then resuspended by sonication and incubated for 15 min in homogenization buffer containing 1% SDS. Following pelleting of insoluble material (16,000g × 15 min), the SDS-extract was electrophoresed on SDS-PAGE (4-12% Bis-Tris polyacrylamide gel from Invitrogen, USA), blotted to PVDF membrane and probed with a 1:1000 dilution of 6E10 (Covance, USA).

### ELISA

Aβ1-42 was quantified in hippocampus and cortex samples using Aβ1-42 ELISA kits (TSZ, USA) in accordance with the instructions of the manufacturer. We have described this procedure in detail in Supplementary Methods.

### Immunohistochemical analysis

Mouse brains were processed for immunohistochemistry using standard protocols. We did not treat the sections with formic acid for immunohistochemical analysis Antibodies used were: rabbit anti-pT181, rabbit anti-pS214, rabbit anti-pS396, rabbit anti-AT8 antibody or rabbit anti-Aβ1-42 antibody (Abcam, UK). AEC system (Zhongshan Goldenbridge Biotechnology, China) was used. We have described this procedure in detail in Supplementary Methods.

### Data analysis

We have analyzed all data using SPSS software, performed one-way or two-way ANOVA with post' hoc test. Differences with a probability level of 95% (*P* > 0.05) were considered significant.

## SUPPLEMENTARY MATERIAL TABLES AND FIGURES



## References

[1] Steen E, Terry BM, Rivera EJ, Cannon JL, Neely TR, Tavares R, Xu XJ, Wands JR, Monte SM (2005). Impaired insulin and insulin-like growth factor expression and signaling mechanisms in Alzheimer's disease-is this type 3 diabetes?. J Alzheimers Dis.

[2] Humpel C (2011). Chronic mild cerebrovascular dysfunction as a cause for Alzheimer's disease?. Exp Gerontol.

[3] Okura T, Langa KM (2011). Caregiver burden and neuropsychiatric symptoms in older adults with cognitive impairment: the Aging, Demographics, and Memory Study (ADAMS). Alzheimer Dis Assoc Disord.

[4] Teri L, Ferretti LE, Gibbons LE, Logsdon RG, McCurry SM, Kukull WA, McCormick WC, Bowen JD, Larson EB (1999). Anxiety of Alzheimer's disease: prevalence, and comorbidity. J Gerontol A Biol Sci Med Sci.

[5] Bornemann KD, Staufenbiel M (2000). Transgenic mouse models of Alzheimer's disease. Ann N Y Acad Sci.

[6] Pugh PL, Richardson JC, Bate ST, Upton N, Sunter D (2007). Non-cognitive behaviours in an APP/PS1 transgenic model of Alzheimer's disease. Behav Brain Res.

[7] Lewis J, McGowan E, Rockwood J, Melrose H, Nacharaju P, Van Slegtenhorst M, Gwinn-Hardy K, Paul Murphy M, Baker M, Yu X, Duff K, Hardy J, Corral A, Lin WL, Yen SH, Dickson DW, Davies P, Hutton M (2000). Neurofibrillary tangles, amyotrophy and progressive motor disturbance in mice expressing mutant (P301L) tau protein. Nat Genet.

[8] Nomura Y, Okuma Y (1999). Age-related defects in lifespan and learning ability in SAMP8 mice. Neurobiol Aging.

[9] Orta-Salazar E, Feria-Velasco A, Medina-Aguirre GI, Diaz-Cintra S (2013). Morphological analysis of the hippocampal region associated with an innate behaviour task in the transgenic mouse model (3xTg-AD) for Alzheimer disease. Neurologia.

[10] Lee KW, Lee SH, Kim H, Song JS, Yang SD, Paik SG, Han PL (2004). Progressive cognitive impairment and anxiety induction in the absence of plaque deposition in C57BL/6 inbred mice expressing transgenic amyloid precursor protein. J Neurosci Res.

[11] Maurer K, Volk S, Gerbaldo H (1997). Auguste D and Alzheimer's disease. Lancet.

[12] Selkoe DJ (1998). The cell biology of beta-amyloid precursor protein and presenilin in Alzheimer's disease. Trends Cell Biol.

[13] Shimura H, Schwartz D, Gygi SP, Kosik KS (2004). CHIP-Hsc70 complex ubiquitinates phosphorylated tau and enhances cell survival. J Biol Chem.

[14] Lu J, Li T, He RQ, Bartlett PF, Götz J (2014). Visualizing the microtubule-associated protein Tau in the nucleus. Sci China Life Sci.

[15] Bartlett PF, He RQ (204). Introduction to the thematic issue “From brain function to therapy”. Sci China Life Sci.

[16] Maloney B, Ge YW, Greig N, Lahiri DK (2004). Presence of a “CAGA box” in the APP gene unique to amyloid plaque-forming species and absent in all APLP-1/2 genes: implications in Alzheimer's disease. FASEB J.

[17] Takeuchi M, Bucala R, Suzuki T, Ohkubo T, Yamazaki M, Koike T, Kameda Y, Makita Z (2000). Neurotoxicity of advanced glycation end-products for cultured cortical neurons. J Neuropathol Exp Neurol.

[18] Sasaki N, Fukatsu R, Tsuzuki K, Hayashi Y, Yoshida T, Fujii N, Koike T, Wakayama I, Yanagihara R, Garruto R, Amano N, Makita Z (1998). Advanced glycation end products in Alzheimer's disease and other neurodegenerative diseases. Am J Pathol.

[19] Wei Y, Chen L, Chen J, Ge L, He RQ (2009). Rapid glycation with D-ribose induces globular amyloid-like aggregations of BSA with high cytotoxicity to SH-SY5Y cells. BMC Cell Biol.

[20] Lu Y, He RQ (2014). GRP75 of CHO Cells Responds to Ribosylation. Prog Biochem Biophys.

[21] Chen L, Wei Y, Wang XQ, He RQ (2010). Ribosylation Rapidly Induces a-Synuclein to Form Highly Cytotoxic Molten Globules of Advanced Glycation End Products. PLoS ONE.

[22] Han C, Lu Y, Wei Y, Liu Y, He RQ (2011). D-ribose induces cellular protein glycation and impairs mouse spatial cognition. PLoS ONE.

[23] Su T, He RQ (2013). The Abnormally High Level of Uric D-Ribose for Type-2 Diabetics. Prog Biochem Biophys.

[24] Su T, He RQ (2014). D-ribose, an overlooked player in type 2 diabetes mellitus?. Sci China Life Sci.

[25] Su T, He RQ (2015). An insight of D-ribose metabolic imbalance in Type 2 diabetes mellitus. Prog Biochem Biophys.

[26] Broom AD, Townsend LB, Jones JW and Robins RK (1964). Purine Nucleosides. Vi. Further Methylation Studies of Naturally Occurring Purine Nucleosides. Biochemistry.

[27] Keller PJ, Le Van Q, Kim SU, Bown DH, Chen HC, Kohnle A, Bacher A, Floss HG (1988). Biosynthesis of riboflavin: mechanism of formation of the ribitylamino linkage. Biochemistry.

[28] Mauser M, Hoffmeister HM, Nienaber C, Schaper W (1985). Influence of ribose, adenosine, and “AICAR” on the rate of myocardial adenosine triphosphate synthesis during reperfusion after coronary artery occlusion in the dog. Circ Res.

[29] Teitelbaum JE, Johnson C, St Cyr J (2006). The use of D-ribose in chronic fatigue syndrome and fibromyalgia: a pilot study. J Altern Complement Med.

[30] Perlmutter NS, Wilson RA, Angello DA, Palac RT, Lin J, Brown BG (1991). Ribose facilitates thallium-201 redistribution in patients with coronary artery disease. J Nucl Med.

[31] Moses AA, Nwamaka L, Nwabudike, Ilesanmi OR (2009). Analgesic, learning and memory and anxiolytic effects of insulin in mice. Behavioural Brain Research.

[32] Strachan MW, Frier BM, Deary IJ (2003). Type 2 diabetes and cognitive impairment. Diabet Med.

[33] Christopher G, MacDonald J (2005). The impact of clinical depression on working memory. Cogn Neuropsychiatry.

[34] Guo M, Lu Y, Garza JC, Li Y, Chua SC, Zhang W, Lu B, Lu XY (2012). Forebrain glutamatergic neurons mediate leptin action on depression-like behaviors and synaptic depression. Transl Psychiatry.

[35] Su JH, Cummings BJ, Cotman CW (1994). Early phosphorylation of tau in Alzheimer's disease occurs at Ser-202 and is preferentially located within neurites. Neuroreport.

[36] Abraha A, Ghoshal N, Gamblin TC, Cryns V, Berry RW, Kuret J, Binder LI (2000). C-terminal inhibition of tau assembly *in vitro* and in Alzheimer's disease. J Cell Sci.

[37] Hoffmann R, Lee VM, Leight S, Varga I, Otvos LJ (1997). Unique Alzheimer's Disease Paired Helical Filament Specific Epitopes Involve Double Phosphorylation at Specific Sites. Biochemistry.

[38] Diniz BS, Pinto JA, Forlenza OV (2008). Do CSF total tau, phosphorylated tau, and beta-amyloid 42 help to predict progression of mild cognitive impairment to Alzheimer's disease? A systematic review and meta-analysis of the literature. World J Biol Psychiatry.

[39] Ahmed N, Ahmed U, Thornalley PJ, Hager K, Fleischer G, Munch G (2005). Protein glycation, oxidation and nitration adduct residues and free adducts of cerebrospinal fluid in Alzheimer's disease and link to cognitive impairment. J Neurochem.

[40] Hong M, Lee VM (1997). Insulin and insulin-like growth factor-1 regulate tau phosphorylation in cultured human neurons. J Biol Chem.

[41] Norelle L. Daly, Ralf Hoffmann, Laszlo Otvos J, Craik DJ (2000). Role of Phosphorylation in the Conformation of Tau Peptides Implicated in Alzheimer's Disease. Biochemistry.

[42] Reaume AG, Howland DS, Trusko SP, Savage MJ, Lang DM, Greenberg BD, Siman R, Scott RW (1996). Enhanced amyloidogenic processing of the beta-amyloid precursor protein in gene-targeted mice bearing the Swedish familial Alzheimer's disease mutations and a “humanized” Abeta sequence. J Biol Chem.

[43] Yang M, Lu J, Miao J, Rizak J, Yang J, Zhai R, Zhou J, Qu J, Wang J, Yang S, Ma Y, Hu X, He RQ (2014). Alzheimer's disease and methanol toxicity (part 1): chronic methanol feeding led to memory impairments and tau hyperphosphorylation in mice. J Alzheimers Dis.

[44] Segal S, Foley J (1958). The metabolism of D-ribose in man. J Clin Invest.

[45] Park CR, Johnson LH, Wright JH, Batsel H (1957). Effect of insulin on transport of several hexoses and pentoses into cells of muscle and brain. Am J Physiol.

[46] Carola V, D'Olimpio F, Brunamonti E, Mangia F, Renzi P (2002). Evaluation of the elevated plus-maze and open-field tests for the assessment of anxiety-related behaviour in inbred mice. Behavioural brain research.

[47] Trento M, Raballo M, Trevisan M, Sicuro J, Passera P, Cirio L, Charrier L, Cavallo F, Porta M (2012). A cross-sectional survey of depression, anxiety, and cognitive function in patients with type 2 diabetes. Acta Diabetol.

[48] Ke YD, Delerue F, Gladbach A, Gotz J, Ittner LM (2009). Experimental diabetes mellitus exacerbates tau pathology in a transgenic mouse model of Alzheimer's disease. PLoS One.

[49] Su T, Xin L, He YG, Wei Y, Song YX, Li WW, Wang XM, He RQ (2013). The Abnormally High Level of Uric D-Ribose for Type-2 Diabetics. Progress in Biochemistry and Biophysics.

[50] Tilson HA, Cabe PA, Mitchell CL (1978). Behavioral and neurological toxicity of polybrominated biphenyls in rats and mice. Environ Health Perspect.

[51] Akanmu MA, Nwabudike NL, Ilesanmi OR (2009). Analgesic, learning and memory and anxiolytic effects of insulin in mice. Behav Brain Res.

[52] Vorhees CV, Williams MT (2006). Morris water maze: procedures for assessing spatial and related forms of learning and memory. Nat Protoc.

[53] Dunn AJ, Swiergiel AH (2005). Effects of interleukin-1 and endotoxin in the forced swim and tail suspension tests in mice. Pharmacol Biochem Behav.

[54] Weng D, Lu Y, Wei Y, Liu Y, Shen P (2007). The role of ROS in microcystin-LR-induced hepatocyte apoptosis and liver injury in mice. Toxicology.

[55] Xie Z, Culley DJ, Dong Y, Zhang G, Zhang B, Moir RD, Frosch MP, Crosby G, Tanzi RE (2008). The common inhalation anesthetic isoflurane induces caspase activation and increases amyloid beta-protein level *in vivo*. Annals of neurology.

