# Supplementary material for: Gavage of D-Ribose induces Aβ-like deposits, Tau hyperphosphorylation as well as memory loss and anxiety-like behavior in mice
Source: Oncotarget. 2015 Oct 7;6(33):34128–42. doi: 10.18632/oncotarget.6021 (PMC4741441; doi:10.18632/oncotarget.6021)
Supplement: Supplementary file 1 [file oncotarget-06-34128-s001.pdf]

# Gavage of D-Ribose induces Aβ-like deposits, Tau hyperphosphorylation as well as memory loss and anxiety-like behavior in mice

**Supplementary Material**

### Tau hyperphosphorylation and Aβ-like deposition is related with anxiety-like behavior

According to Ferretti and coworkers, anxiety can cause memory problems [1]. Much work indicates that the anxiety-like behavior has relationship with Tau hyperphosphorylation and Aβ-like deposition. The reasons are as follows. First, Aβ deposit is correlated with dementia such as anxiety [2-4]. Second, Tau hyperphosphorylation and Aβ-like deposition in the brain are associated with memory loss and anxiety-like phenotypes [5, 6]. Third, as described by Nelson and colleagues [7], behavioral abnormalities in AD including anxiety can result from the dysfunction and degeneration of neurons in brain regions involved in cognition and mood such as the hippocampus, associated cortical regions and amygdala. Fourth, according to Melrose and colleagues, Tau protein abnormalities, e.g. phosphorylation at Ser202 (AT8 epitope) was significantly increased in the hippocampus related with anxiety phenotypes in the particular transgenic mouse model [5]. Finally, for D-Ribose-gavaged mice, both Aβ-like deposition and Tau hyperphosphorylation were observed, especially in the hippocampus. These data imply that Tau hyperphosphorylation and Aβ-like deposition are correlated with the decline in spatial learning and memory might as well as the anxiety-like behavior in D-Ribose-gavaged mice.

### Oral administration of D-Ribose can be used as an animal model for cognitive impairment and diabetic encephalopathy

Our data suggest that long-term D-Ribose gavaged mice could be employed as a model for age-related cognitive impairment and diabetic encephalopathy. This view is based on the following observations: (1) D-Ribose abundantly exists in the human body [8, 9] and diabetic patients have an abnormally high level of uric D-Ribose [10]; (2) Type 2 diabetes mellitus is not only characterized by a chronic imbalance of D-Glucose metabolism, but also of D-Ribose

metabolism [11]. (3) D-Ribose and ribosylated proteins are more cytotoxic than D-Glucose and its glycated products both in vivo and in vitro [12]; (4) Alzheimer's disease is regarded as the 'type 3 diabetes' which is correlated with imbalance of energetic metabolism [13]; (5) oral administration of D-Ribose induces memory loss and anxiety-like behavior, similar to the symptoms of AD patients; (6) D-Ribose-gavaged mice display AD-like symptoms without abnormal phenotypes of altered fasting blood D-Glucose levels, glycation (AGEs), increased body weight, reduced muscle strength, and impaired motor ability and co-ordination; (7) according to the definition of AD which is an age-related acquired disease except for very rare incidence of the genetic frontal-temporal dementia [14], risk factors in vivo and in vitro are important in the progression of AD, for instance an imbalance in energy and substance metabolism [15]; and finally, (8) oral administration of D-Ribose to mice, rats and other mammals is very conveniently performed with a highly reproducible phenotype.

## SUPPLEMENTARY METHODS

### Gavage procedure

The gavage procedure was performed by professional workers in the animal house of Institute of Biophysics, Chinese Academy of Sciences. Grab the skin over the mouse shoulder firmly with the thumb and middle fingers, stretch the head and neck with the index finger to make the esophagus straight. Direct the ball-tip of the feeding needle along the roof of the mouth and toward the right side of the back of the pharynx, then gently pass down into the esophagus and inject the solution. No resistance should be felt. When being sacrificed, mice were first anesthetized with 10% chloral hydrate and then perfused through heart with at least 50 ml saline per one mouse, for eliminating blood from organs. After that, the organs were fetched out. All processes were carried out according to the Animal Welfare and Research Ethics Committee of the Institute of Biophysics, Chinese Academy of Sciences (Permit number: SYXK2013-77).

**Gel electrophoresis and Western blotting/dot blotting**

Levels of AGEs in hippocampus, cortex, liver, kidney and serum were determined by Western blotting. The same method was used to analyze the expression of phosphorylated T181 (pT181), S214 (pS214), S396 (pS396), and Ser199/202 (AT8), or dephosphorylated Ser199/202 (Tau-1) (recognizing aminoacid residues 189-207), Tau-5 (total Tau), APP and β-actin in hippocampus and cortex tissues of mice treated with D-Ribose or D-Glucose at different concentrations. Tissues were lysated using lysate buffer (P0013, beyotime, China) according to manufacturer's instruction. Protein concentrations were quantified with the BCA Protein Assay Kit (Pierce, Rockford, IL, USA). Equivalent amounts of protein (20–30 μg) were resolved on 12% SDS-PAGE gels and transferred to PVDF membranes. Membranes were then incubated, respectively, with anti-AGE monoclonal antibody 6D12 (TransGenic), anti-pT181 polyclonal antibody (SAB), anti-pS214 polyclonal antibody (Invitrogen), anti-pS396 polyclonal antibody (Invitrogen), anti-AT8 polyclonal antibody (Invitrogen), anti-Tau-1 monoclonal antibody (Millipore), anti-Tau-5 monoclonal antibody (Millipore), anti-APP polyclonal antibody (CST) or anti-β-actin monoclonal antibody (Sigma) overnight at 4°C. Each membrane was washed three times with PBS with 0.1% (v/v) Tween-20 (PBST, pH 7.4), then incubated with horseradish peroxidase-conjugated anti-mouse or anti-rabbit IgG at 37°C for 1 h. The membranes were again washed three times with PBST, and then immunoreactive bands were visualized using enhanced chemiluminescence detection reagents (Applygen, China). The protein bands were visualized after exposure of the membranes to Kodak X-ray film and quantified by Quantity One 1D analysis software 4.5.2 (Bio-Rad, Hercules, USA). The expression of Aβ1-42 in hippocampal and cortical tissues were determined by dot blotting. Here, equivalent amounts of protein (4-6 μg) were directly spotted onto the nitrocellulose membrane which was allowed to dry. The membrane was then incubated with an anti-Aβ1-42 polyclonal antibody (CST) overnight at 4°C. The subsequent steps were as done for Western blotting.

For dot blotting, 5 μl of each tissue lysate was spotted to the NC membrane. After the membrane was air-dried for about 10 minutes, it was incubated with 5% skimmed milk, followed by the same procedure as Western blotting.

## ELISA

Hippocampus and cortex tissues were first lysated using lysate buffer (P0013, beyotime, China). Protein concentrations were quantified with the BCA Protein Assay Kit (Pierce, Rockford, IL, USA). Aβ1-42 was quantified in hippocampus and cortex samples using Aβ1-42 ELISA kits (TSZ, USA) in accordance with the instructions of the manufacturer. Briefly, the samples were added to the wells precoated with purified mouse Aβ1-42 antibody. After incubation for 30 min at 37°C, washing buffer was added to every well, still for 30s then drain, repeat 5 times, dry by pat. Then add HRP-conjugate reagent to each well, incubate for 30 min at 37°C. After washing for 5 times, TMB substrate solution was used to develop color for 15 min at 37°C. At last, stop solution was added to each well to stop the reaction (the blue color change to yellow color). Read absorbance at 450nm after adding stop solution and within 15min. Calculate the Aβ1-42 concentration according to standard curve.

## Immunohistochemicaly analysis

Immunohistochemical staining for pT181, pS214, pS396, AT8 and Aβ 1-42 was performed as described. Mice brains were immersed in 4% paraformaldehyde for 48 h immediately after they were dissected. After fixation, brains were embedded in paraffin blocks. Five-μm thick sections were processed for immunohistochemical analyses. Deparaffinized and rehydrated sections were incubated in Target Retrieval Solution at 95°C for 30 min for enhancement of immunoreactivity and then permeabilized with 0.3% $H_2O_2$ in absolute methanol for 10 min to block endogenous peroxidase, followed by incubation in 10% normal goat serum in PBS at room temperature for 30 min. The specimens were incubated overnight at 4°C with anti-pT181, anti-pS214 anti-pS396, AT8 or anti-Aβ1-42 antibody (Abcam, UK) solution diluted in PBS. After washing with PBS, sections were incubated with biotin-labeled secondary antibodies (37°C, 1 h). The immunoreaction was detected using horseradish peroxidase-labeled antibodies (37°C, 1 h) and red staining was visualized with an AEC system (Zhongshan Goldenbridge Biotechnology, China). Slides were lightly counterstained with Harris' haematoxylin and mounted with GVA aqueous mounting medium (Zhongshan Goldenbridge Biotechnology, China) for observation by light microscopy (Nikon Optical, Japan).

**Detection of brain D-Ribose and D-Glucose in brain by UV-HPLC**

D-Ribose and D-Glucose were measured as described previously [16]. The brain was quickly dissected out and 0.1g brain was homogenized in 1 ml lysis buffer immediately. Then, 3 ml acetonitrile was added and vortexed vigorously for 30 s before centrifugation (12,000 rpm, 4°C, and 10 min). The supernatant was layered and 0.4 ml lower aqueous phase was pipetted into 1.5 ml Eppendorf tubes and mixed with 0.6 ml 4-(3-Methyl-5-oxo-2-pyrazolin-1-yl) benzoic acid (MOPBA, final concentration 150 mM, in 250 mM NaOH in 50% methanol-water solution). Samples were vortexed vigorously for 30 s before centrifugation (12,000 rpm, 4°C, and 10 min) and then heated in a 70°C water bath for 90 min, followed by additional centrifugation (12,000 rpm, 4°C, and 10 min). The mixture was acidified by adding 150 μl of an aqueous 2 M HCl solution to precipitate the excess MOPBA. The mixture was vigorously vortexed and then centrifuged (12,000 rpm, 4°C, and 10 min), and then filtrated (0.22 μm). 20 μl of the solution was then subjected to high-performance liquid chromatography (HPLC).

The HPLC system (LC-20A, Shimadzu, Japan) was equipped with an ultraviolet detector. The MOPBA-sugar derivative was collected from the C18 column with a binary mobile phase gradient. Mobile phase A was 10 mM of sodium 1-hexanesulfonate; the pH was stabilized at 2.5 by phosphoric acid. Mobile phase B was 50% acetonitrile solution. The elution conditions were 38%-60% B for 15 min, 100% B for 5 min and 38% for 5 min. The flow rate was 1 ml/min and the column temperature was 40°C. The procedure for D-Glucose analysis was the same procedure for detecting D-Ribose, except in the latter procedure, elution conditions were 42%-60% B for 15 min, 100% B for 5 min and 42% for 5 min, and 20 μl of the solution was injected into the analytical column. The reference concentrations of D-Ribose and D-Glucose were determined according to the standard curve.

# REFERENCES


1. Ferretti L, McCurry SM, Logsdon R, Gibbons L, Teri L. Anxiety and Alzheimer's disease. J Geriatr Psychiatry Neurol. 2001; 14:52-58.
2. Judit E, Lydia GL, Jorge V, Alfredo M, Alberto R, José RA, Frank M, LaFerla, Carlos AS. Intraneuronal beta-amyloid accumulation in the amygdala enhances fear and anxiety in Alzheimer's disease transgenic mice. Biol Psychiatry. 2010; 15:513-21.
3. Zare N, Khalifeh S, Khodagholi F, Shahamati SZ, Motamedi F, Maghsoudi N. Geldanamycin Reduces Aβ-Associated Anxiety and Depression, Concurrent with Autophagy Provocation. J Mol Neurosci. 2015; 10.1007.
4. Pietrzak RH, Lim YY, Neumeister A, Ames D, Ellis KA, Harrington K, Lautenschlager NT, Restrepo C, Martins RN, Masters CL, Villemagne VL, Rowe CC, Maruff P. Amyloid-β, anxiety, and cognitive decline in preclinical Alzheimer disease: a multicenter, prospective cohort study. JAMA Psychiatry. 2015; 72:284-91.
5. Melrose HL, Dachsel JC, Behrouz B, Lincoln SJ, Yue M, Hinkle KM, Kent CB, Korvatska E, Taylor JP, Witten L, Liang YQ, Beevers JE, Boules M, Dugger BN, Serna VA, Gaukhman A, Yu X, Castanedes-Casey M, Braithwaite AT, Ogholikhan S, Yu N, Bass D, Tyndall G, Schellenberg GD, Dickson DW, Janus C, Farrer MJ. Impaired dopaminergic neurotransmission and microtubule-associated protein tau alterations in human LRRK2 transgenic mice. Neurobiol Dis. 2010; 40:503-517.
6. Lavretsky H, Siddarth P, Kepe V, Ercoli LM, Miller KJ, Burggren AC, Bookheimer SY, Huang SC, Barrio JR, Small GW. Depression and anxiety symptoms are associated with cerebral FDDNP-PET binding in middle-aged and older nondemented adults. Am J Geriatr Psychiatry. 2009; 17:493-502.
7. Nelson RL, Guo Z, Halagappa VM, Pearson M, Gray AJ, Matsuoka Y, Brown M, Martin B, Iyun T, Maudsley S, Clark RF, Mattson MP. Prophylactic treatment with paroxetine ameliorates behavioral deficits and retards the development of amyloid and tau pathologies in 3xTgAD mice. Exp Neurol. 2007; 205:166-176.
8. Seuffer R. A new method for the determination of sugars in cerebrospinal fluid (author's transl). J Clin Chem Clin Biochem. 1977; 15:663-668.
9. Cai Y, Liu J., Shi Y, Liang L, Mou S. Determination of several sugars in serum by high-performance anion-exchange chromatography with pulsed amperometric detection. J Chromatogr A. 2005; 1085:98–103.
10. Su T, Xin L, HE YG, Wei Y, Song YX, Li WW, Wang XM, He RQ. The Abnormally High Level of Uric D-Ribose for Type-2 Diabetics. Progress in Biochemistry and Biophysics. 2013; 40:816-825.
11. Keller PJ, Le Van Q, Kim SU, Bown DH, Chen HC, Kohnle A, Bacher A, Floss HG. Biosynthesis of riboflavin: mechanism of formation of the ribitylamino linkage. Biochemistry. 1988; 27:1117-1120.
12. Chen L, Wei Y, Wang XQ, He RQ. Ribosylation Rapidly Induces a-Synuclein to Form Highly Cytotoxic Molten Globules of Advanced Glycation End Products. PLoS ONE. 2010; 5:e9052.
13. Steen E, Terry BM, Rivera EJ, Cannon JL, Neely TR, Tavares R, Xu XJ, Wands JR, Monte SM. Impaired insulin and insulin-like growth factor expression and signaling mechanisms in Alzheimer’s disease-is this type 3 diabetes? J Alzheimers Dis. 2005; 7:63-80.
14. Blennow K, de Leon MJ, Zetterberg H. Alzheimer's disease. Lancet. 2006; 368:387-403.
15. Saraiva AA, Borges MM, Madeira MD, Tavares MA, Paula-Barbosa MM. Mitochondrial abnormalities in cortical dendrites from patients with Alzheimer's disease. J Submicrosc Cytol. 1985; 17:459-464.
16. Castells CB, Arias VC, Castells RC. Precolumn Derivatization of Reducing Carbohydrates with 4 (3-Methyl-5-oxo-2-pyrazolin- 1-yl) Benzoic Acid. Study of Reaction, High-Performance Liquid Chromatographic Separation and Quantitative Performance of Method. Chromatographia. 2002; 56:153-160.

**Supplementary Table 1: Changes in the body weight of mice during the administration of sugars.** Conditions for the treatment were the same as those given in Figure 1. Body weight per month (including pre-gavage) during the treatment is shown in grams. All values are expressed as mean±S.E.M.

| | Control (n=12) | Rib (n=12) 0.375 g/kg·d | Rib (n=12) 3.75 g/kg·d | Glc (n=12) 0.45 g/kg·d | Glc (n=12) 4.5 g/kg·d |
|---|---|---|---|---|---|
| **Pre-gavage** | 21.13±0.14 | 20.59±0.40 | 21.31±0.32 | 20.95±0.12 | 21.31±0.2 |
| **1 mon** | 24.51±0.42 | 25.28±0.53 | 24.47±0.28 | 25.01±0.61 | 24.74±0.4 |
| **2 mon** | 25.16±0.54 | 25.48±0.39 | 25.76±0.39 | 25.60±0.75 | 24.89±0.41 |
| **3 mon** | 29.03±0.69 | 28.35±0.61 | 28.00±0.68 | 28.26±0.80 | 27.19±0.49 |
| **4 mon** | 28.8±0.81 | 27.91±0.50 | 27.64±0.81 | 28.19±0.80 | 27.24±0.42 |
| **5 mon** | 29.74±0.86 | 28.94±0.63 | 28.16±0.91 | 29.39±0.93 | 28.22±0.57 |
| **6 mon** | 31.54±0.31 | 31.40±0.69 | 31.15±0.48 | 31.2±0.90 | 30.36±0.51 |

**Supplementary Table 2: Changes in blood sugar concentrations during treatment.** Mice were gavaged with D-Ribose or D-Glucose for 6 months as described in Figure 1. Fasting blood sugar levels (mM) were determined every month throughout the treatment. All values are expressed as mean±S.E.M.

| | Control (n=12) | Rib (n=12) 0.375 g/kg·d | Rib (n=12) 3.75 g/kg·d | Glc (n=12) 0.45 g/kg·d | Glc (n=12) 4.5 g/kg·d |
|---|---|---|---|---|---|
| **1 mon** | 7.46±0.40 | 6.79±0.51 | 6.99±0.30 | 6.77±0.25 | 7.58±0.22 |
| **2 mon** | 6.70±0.32 | 5.35±0.39 | 5.70±0.34 | 5.14±0.31 | 5.57±0.34 |
| **3 mon** | 9.17±0.50 | 6.42±0.50 | 7.55±0.45 | 6.79±0.51 | 7.95±0.60 |
| **4 mon** | 5.75±0.26 | 5.03±0.18 | 5.48±0.28 | 5.27±0.28 | 5.23±0.24 |
| **5 mon** | 5.37±0.25 | 4.56±0.21 | 4.79±0.26 | 4.49±0.21 | 4.64±0.23 |
| **6 mon** | 9.03±0.45 | 6.68±0.51 | 7.14±0.27 | 6.70±0.31 | 6.84±0.30 |

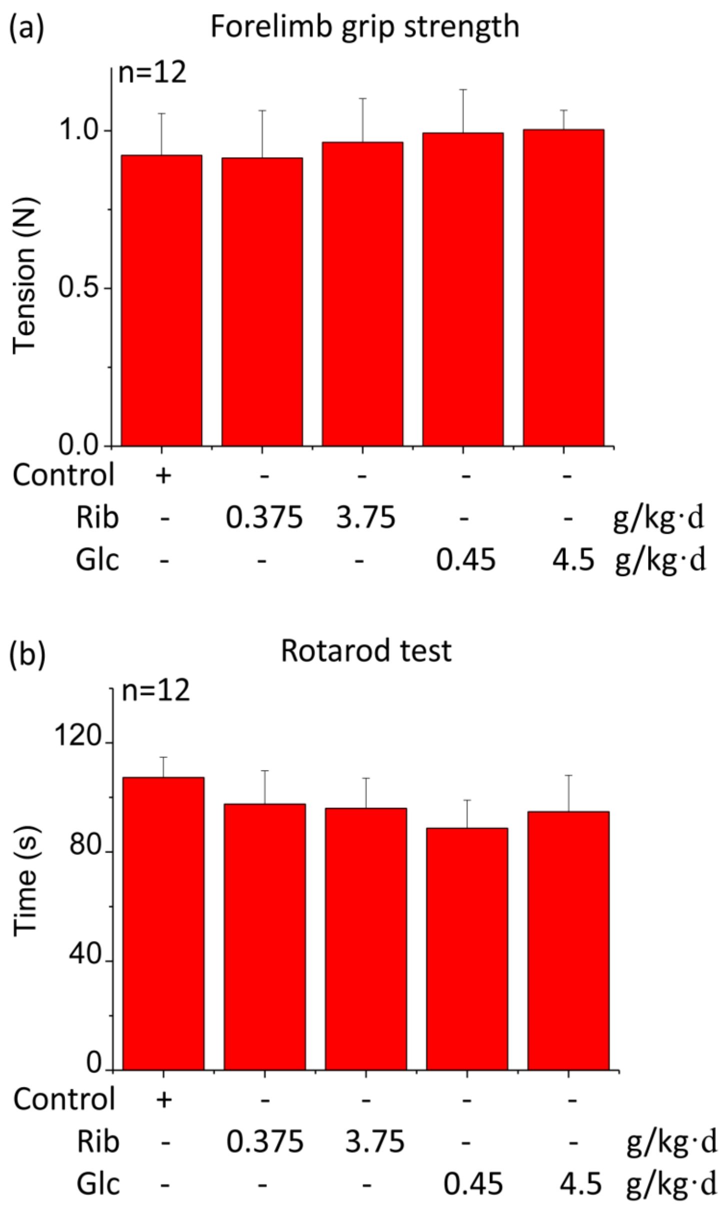


**Supplementary Figure 1: Sugar-treated mice show a normal movement.** Conditions for the sugar treatment were the same as those given in Figure 1. The strength of muscle was determined by the tensile test (a) and motor coordination using the Rotarod (b) which revealed no significantly different between the Ribose- and Glucose-treated mice. All values are expressed as mean±S.E.M.

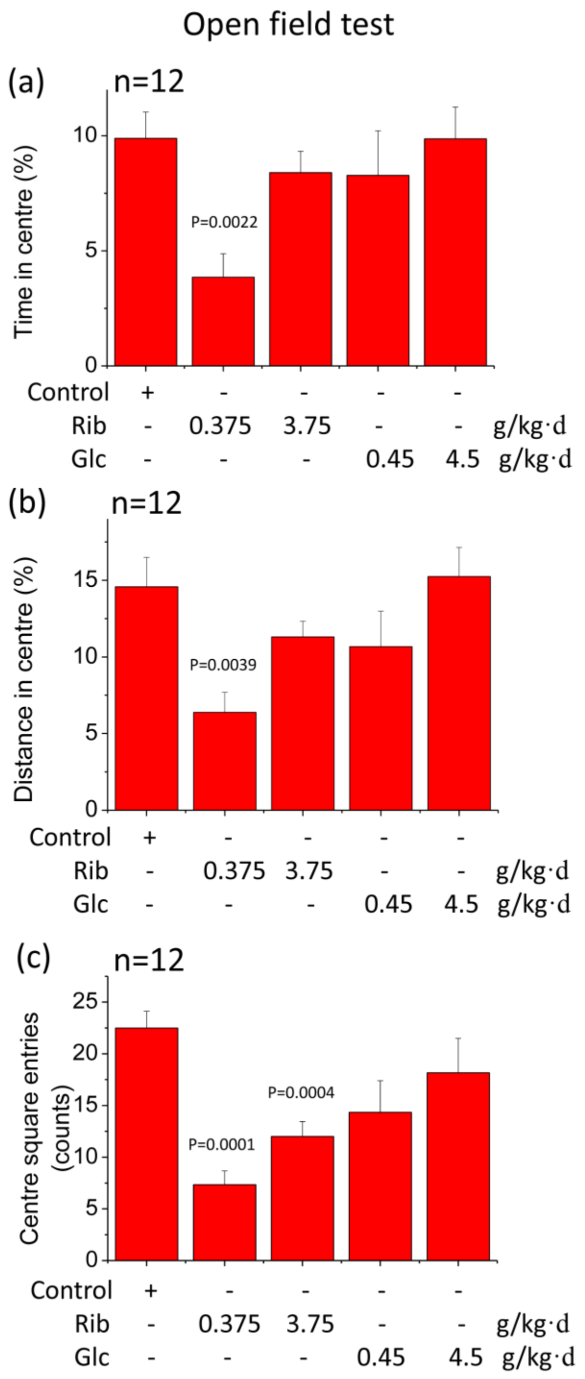


**Supplementary Figure 2: Open field test.** Mice were gavaged with D-Ribose or D-Glucose for 6 months as described in Figure 1. In an open field arena, the percentage of time spent in the centre (a), the percentage of the time travelled in the centre (b) and the number of centre square entries (c) is considered as an index of anxiety. All values are expressed as mean±S.E.M. The P values are obtained from comparative analysis of indicated group with the controls.

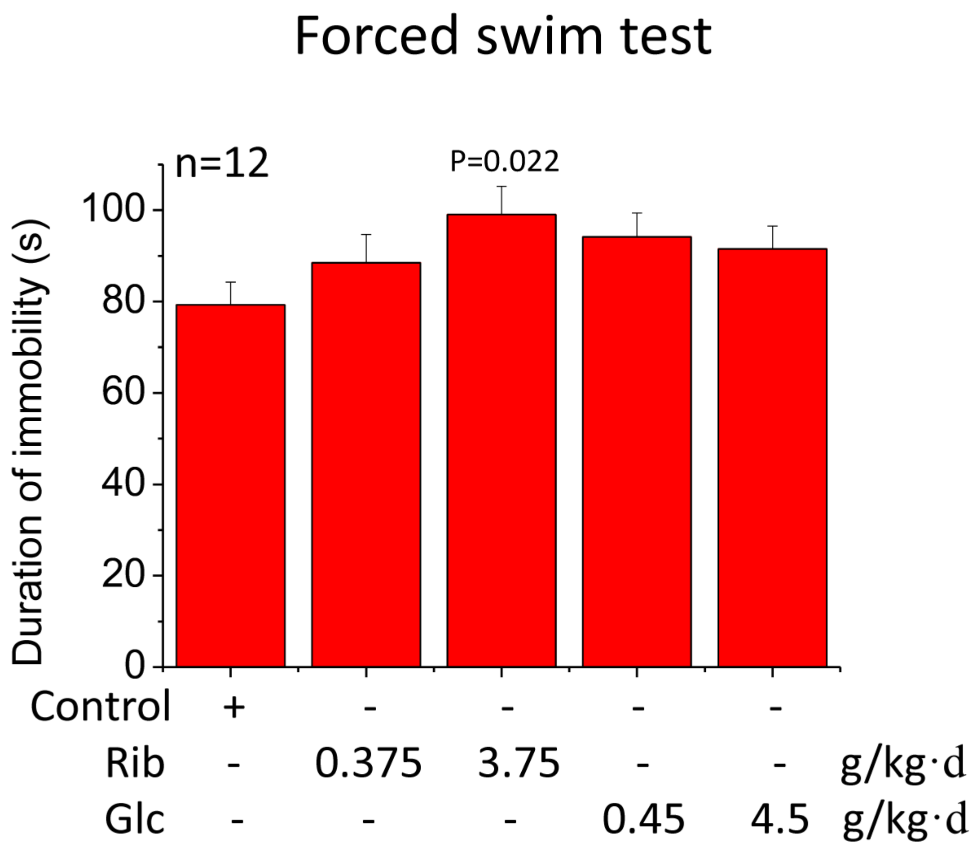


**Supplementary Figure 3: Forced swim test.** Conditions for the treatment were the same as those given in Figure 1. Immobility time was measured during the last 4 min of the 6-min test period in forced swim test. All values are expressed as mean±S.E.M. The P value is obtained from comparative analysis of indicated group with the control.

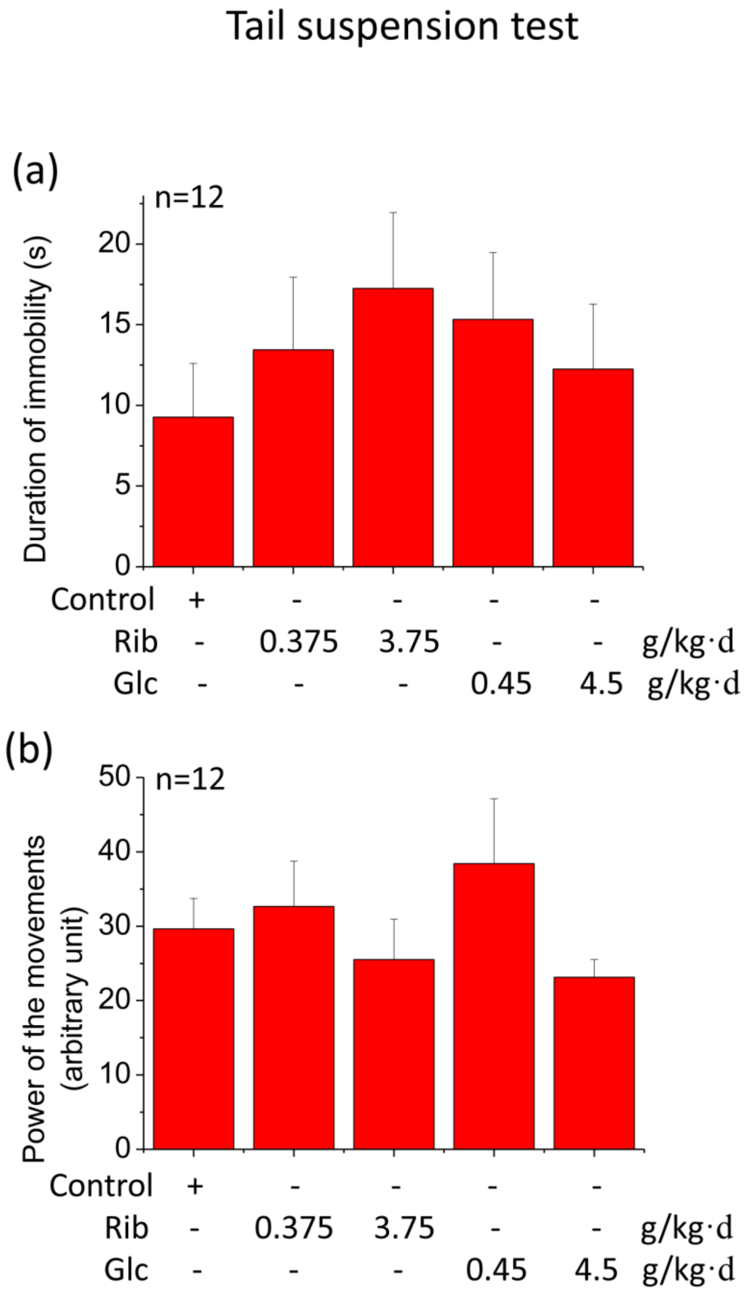


**Supplementary Figure 4: Tail suspension test.** Conditions for the treatment were the same as those given in Figure 1. In the tail suspension test, the duration of immobility (a) and power of the movement (b) was measured during a 6-min test period.

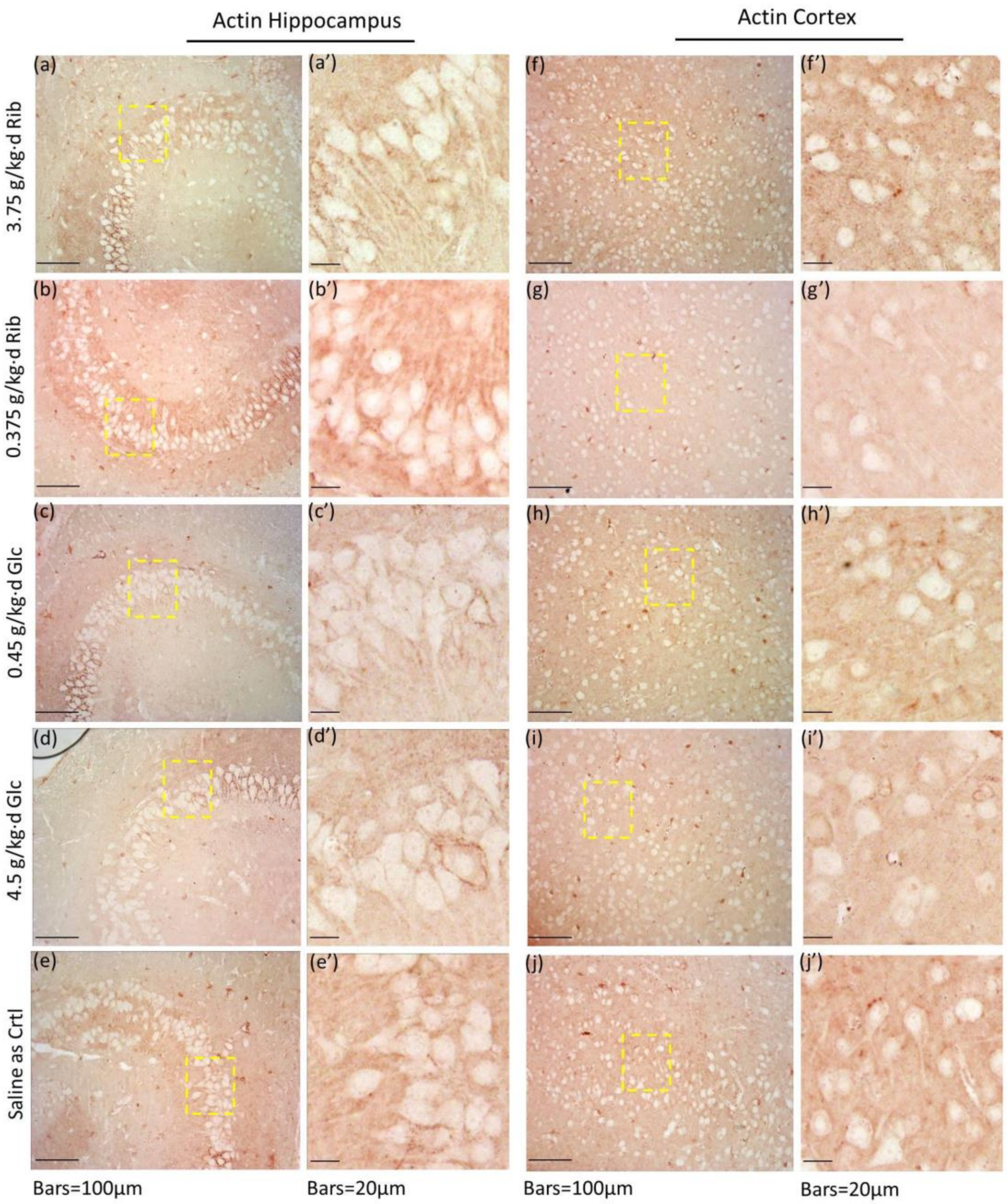


**Supplementary Figure 5: Immunohistochemical staining of actin in hippocampus and cortex.** Conditions for the treatment were the same as those given in Figure 5. β-Actin in the hippocampus (a,a'-e,e') and cortex (f,f'-j,j') detected by immunohistochemistry using anti-β-actin monoclonal antibody.

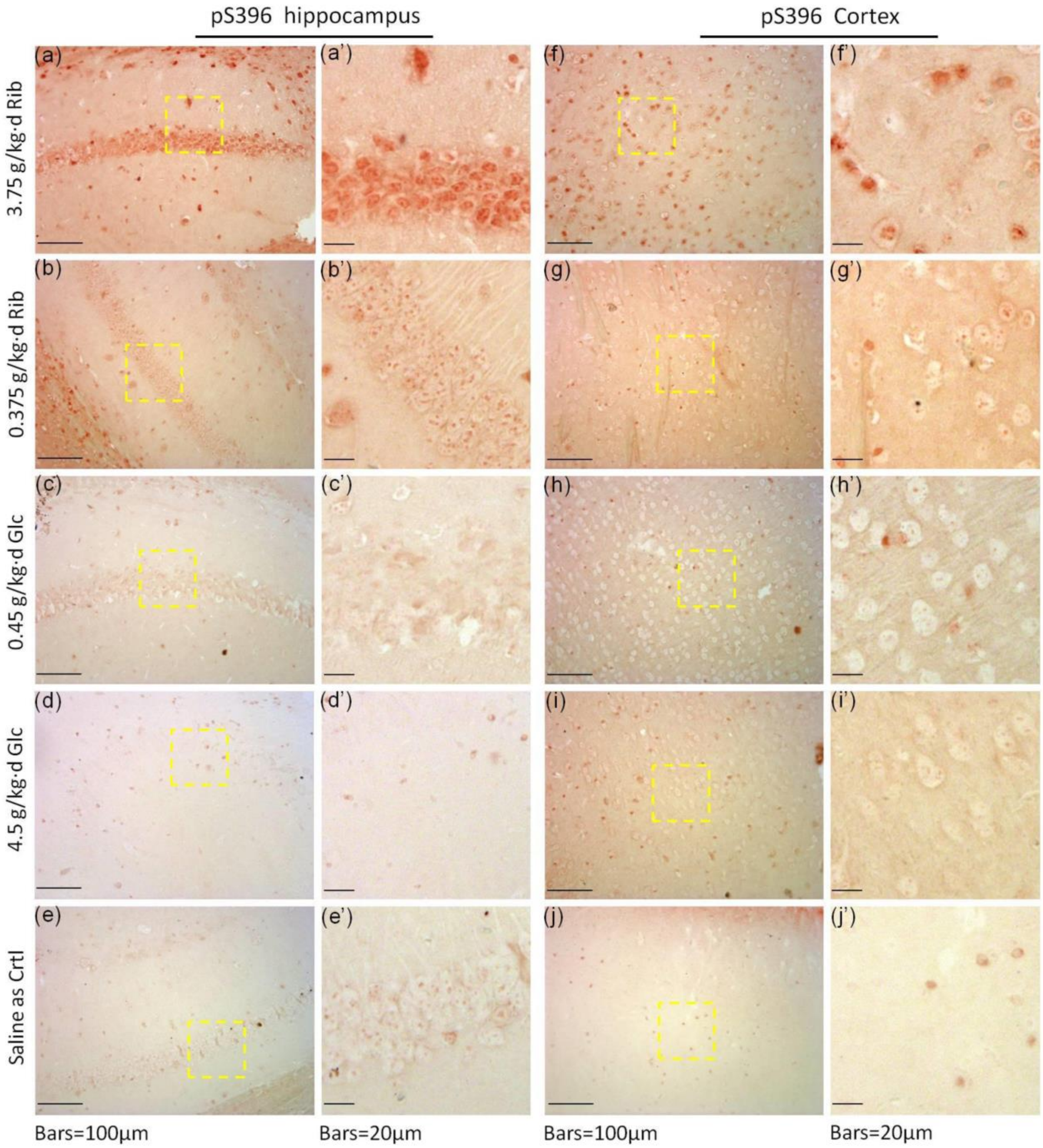


**Supplementary Figure 6: Immunohistochemical staining of pS396 in the hippocampus and cortex.** Conditions for the treatment were the same as those given in Figure 5. pS396 in the hippocampus (a,a'-e,e') and cortex (f,f'-j,j') detected by immunohistochemistry using anti-Tau pS396 polyclonal antibody.

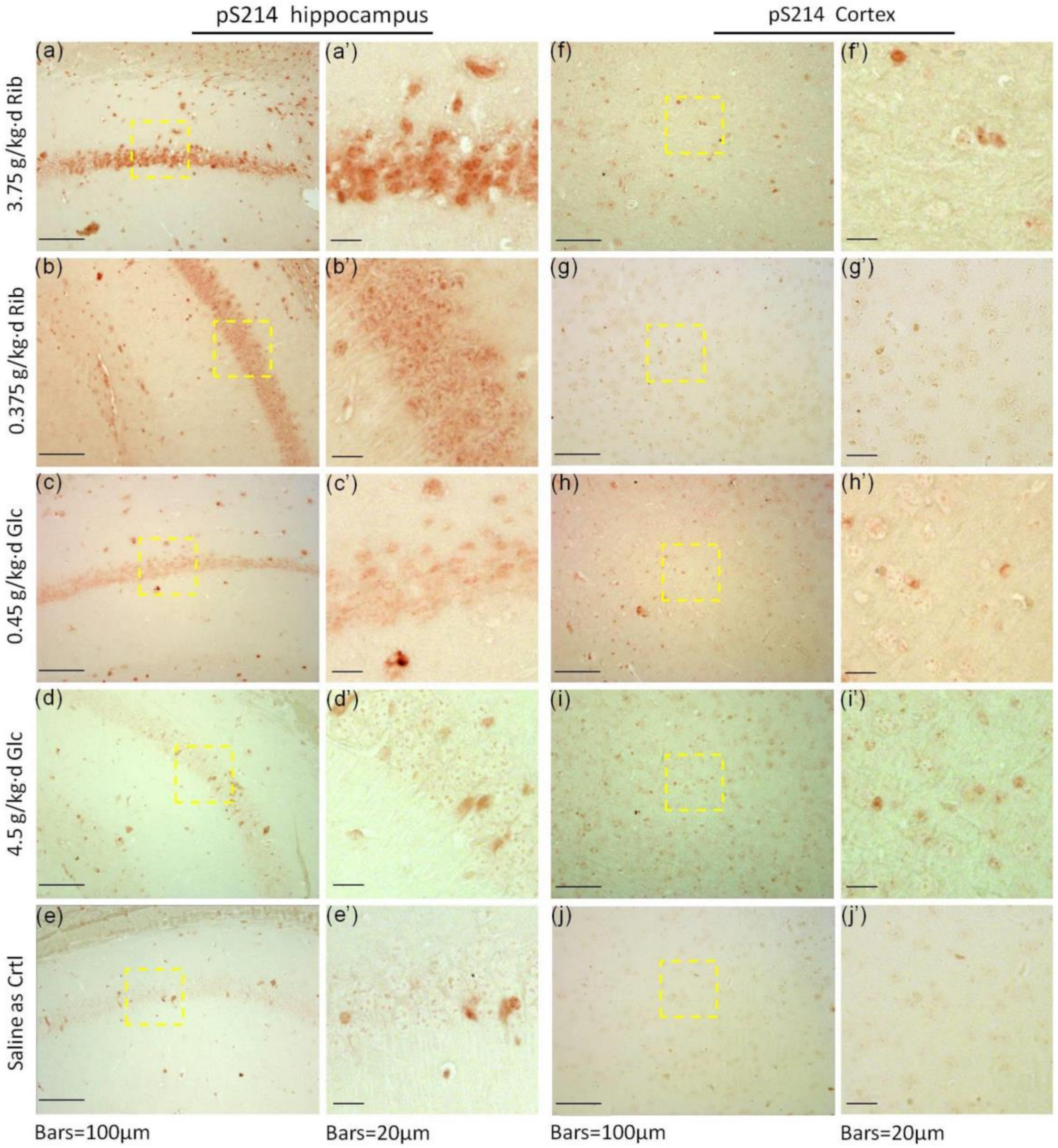


**Supplementary Figure 7: Immunohistochemical staining of pS214 in hippocampus and cortex.** Conditions for the treatment were the same as those given in Figure 5. pS214 in the hippocampus (a,a'-e,e') and cortex (f,f'-j,j') detected by immunohistochemistry using anti-Tau pS214 polyclonal antibody.

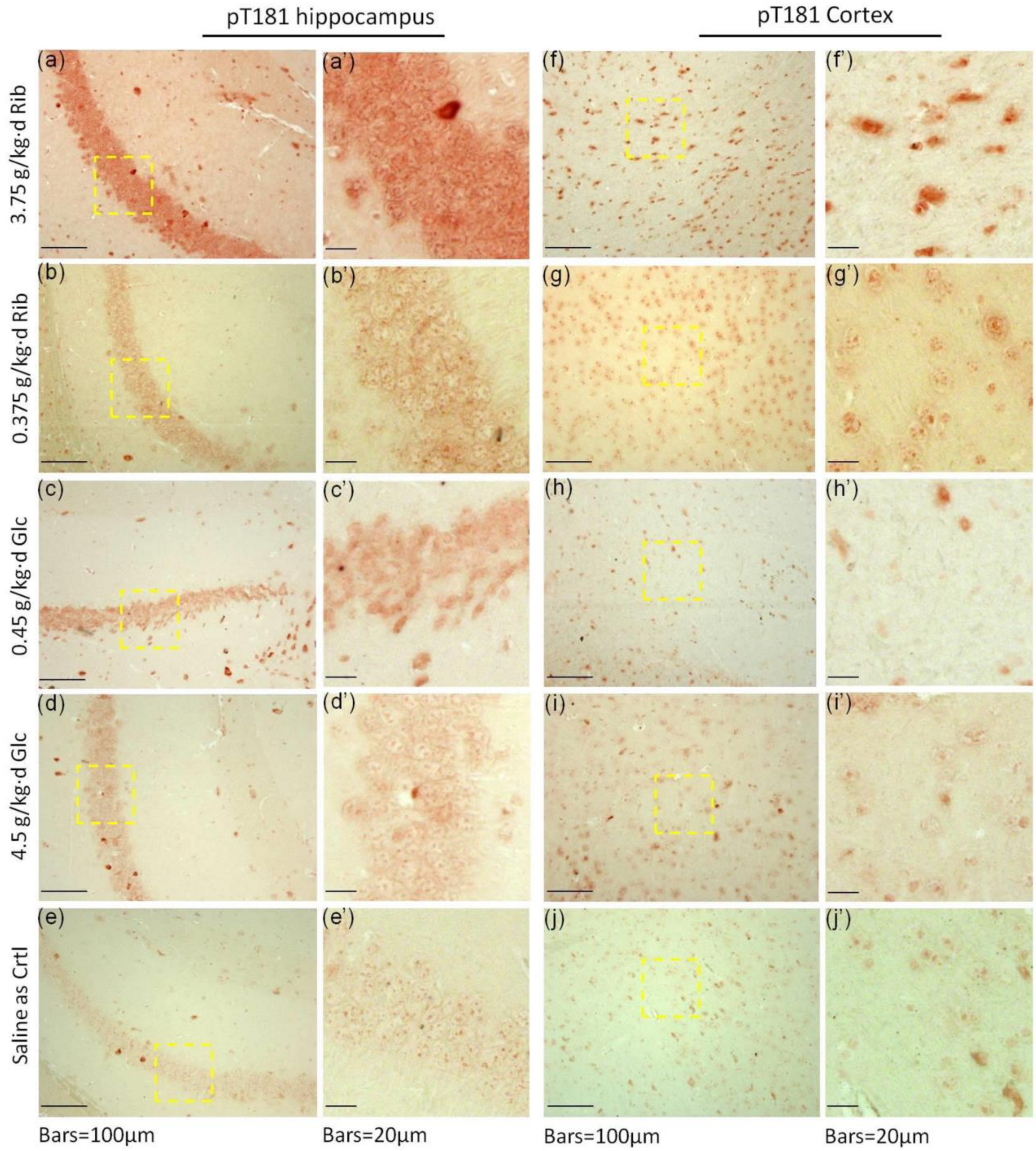


**Supplementary Figure 8: Immunohistochemical staining of pT181 in hippocampus and cortex.** Conditions for the treatment were the same as those given in Figure 5. pT181 in the hippocampus (a,a'-e,e') and cortex (f,f'-j,j') detected by immunohistochemistry using anti-Tau pT181 polyclonal antibody.

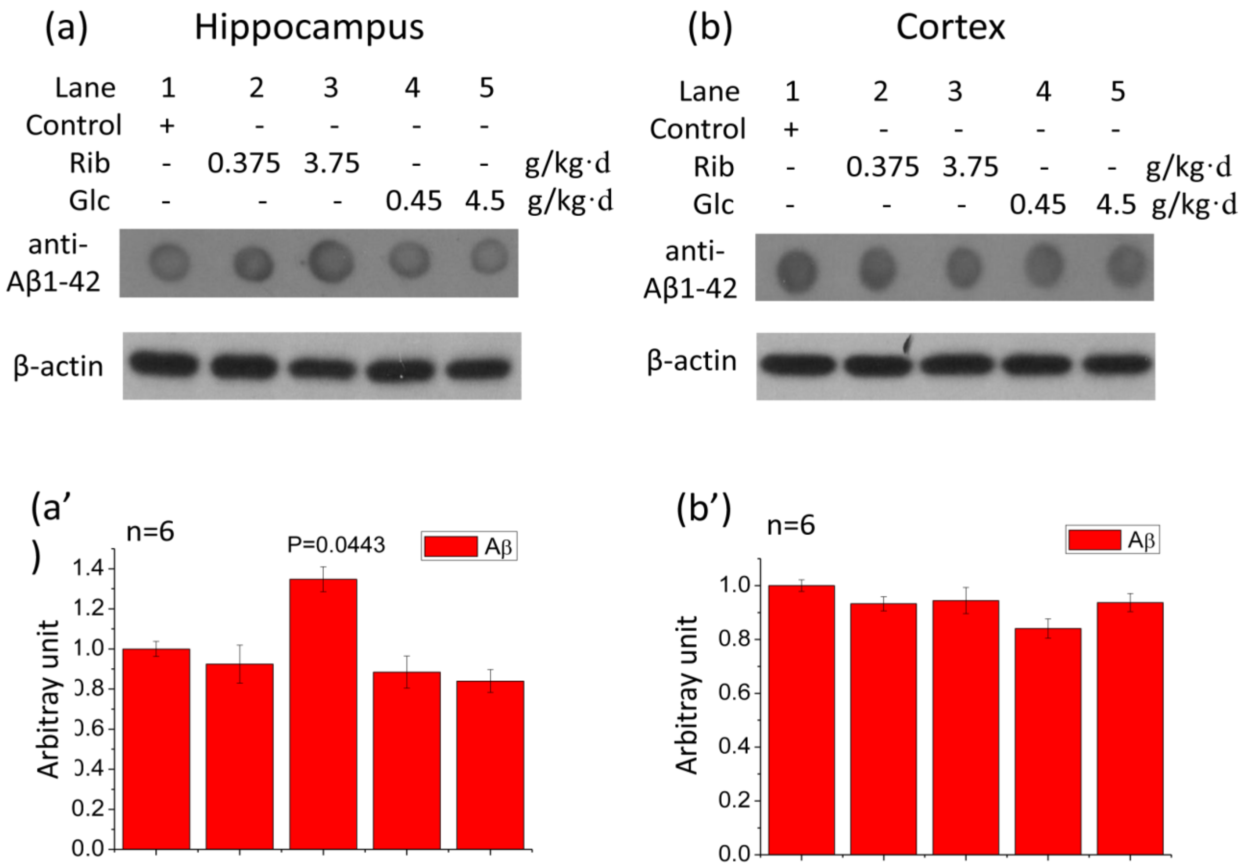


**Supplementary Figure 9: Detection of amyloid-β (Aβ) in brain by Dot blotting.** Conditions for the treatment were the same as those given in Figure 6, except that the expression of Aβ1-42 was detected by Dot blotting using anti-Aβ1-42 polyclonal antibody. β-Actin was used as a loading control. Quantification is shown in a' and b'. The saline control value was set as 1.0. All values are expressed as mean±S.E.M. The P value is obtained from comparative analysis of indicated group with the control.

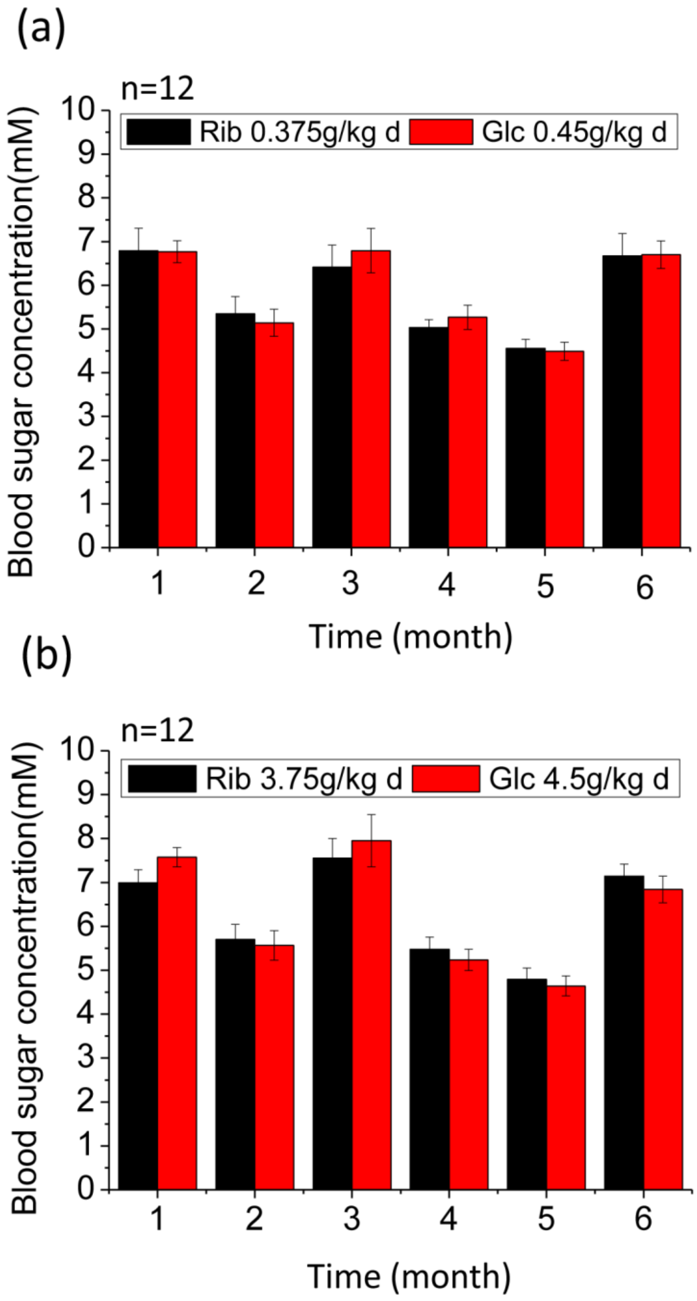


**Supplementary Figure 10: Changes in blood sugar levels between D-Ribose- and D-Glucose-treated mice recorded monthly.** Mice were gavaged with D-Ribose, D-Glucose, and saline as a control, respectively, as shown in Figure 1. (a) Blood sugar levels in mice treated with D-Ribose (0.375 g/kg·d) or D-Glucose (0.45 g/kg·d). (b) Blood sugar levels (mM) in mice treated with D-Ribose (3.75 g/kg·d) or D-Glucose (4.5 g/kg·d). All values are expressed as mean±S.E.M.

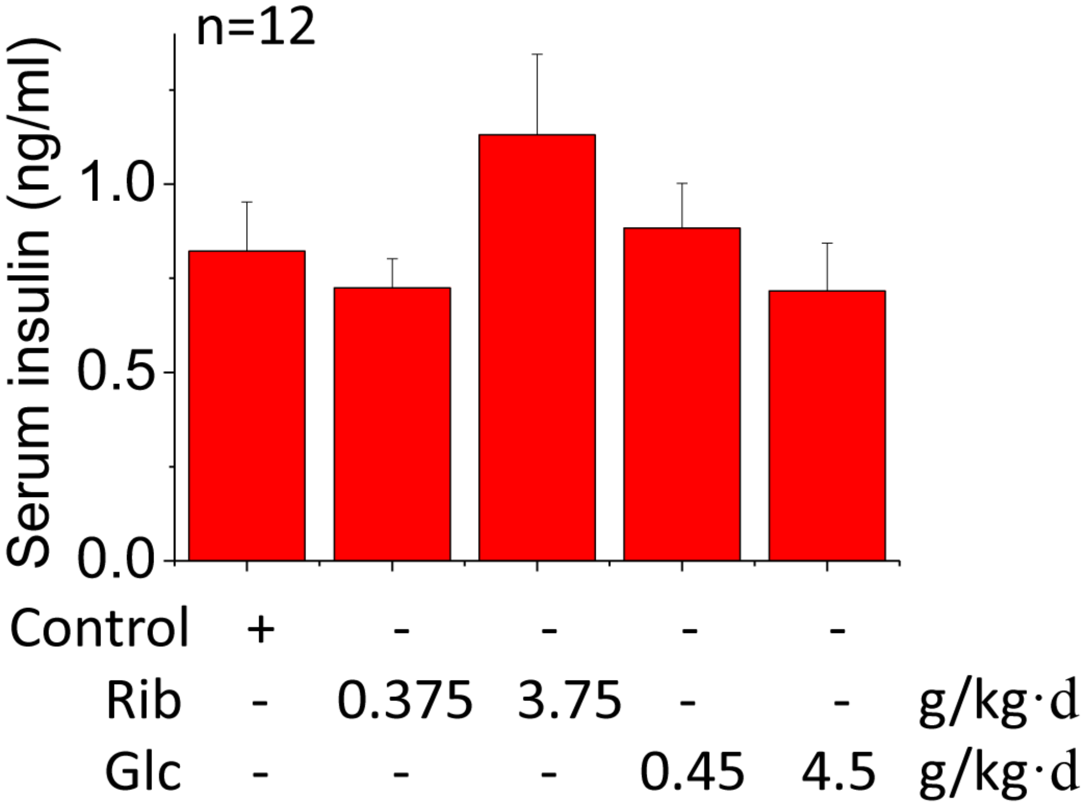


**Supplementary Figure 11: Levels of insulin in blood.** Conditions for the treatment were the same as those given in Figure 1. The blood insulin concentration was measured by radioimmunoassay.

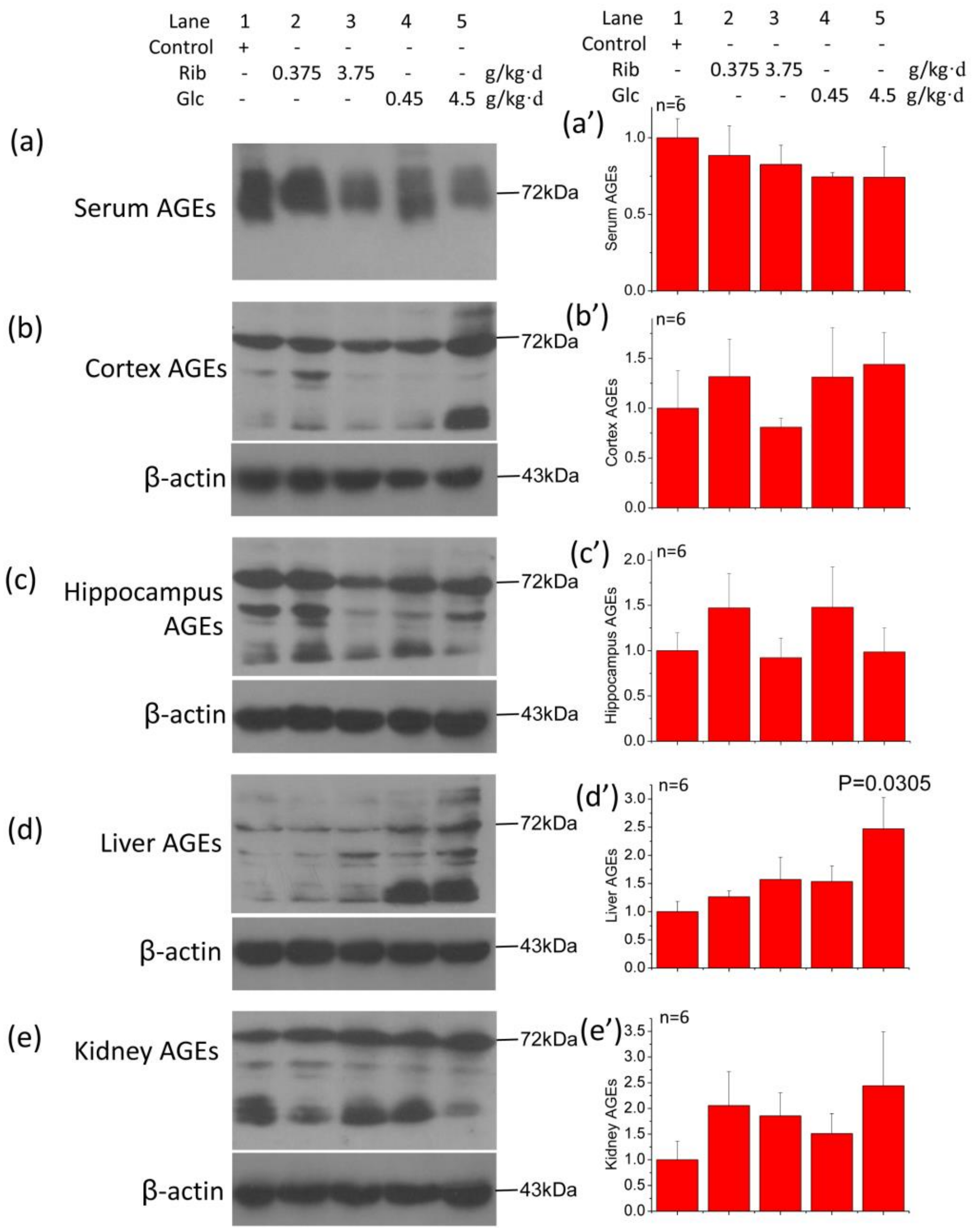


**Supplementary Figure 12: Changes in levels of AGEs in mouse serum, cortex, hippocampus, liver and kidney.** Conditions for the treatment were the same as those given in Figure 1. AGEs in serum (a), cortex (b), hippocampus (c), liver (d), and kidney (e) detected by Western blotting with an anti-AGEs 6D12 monoclonal antibody. β-Actin was used as a loading control except for serum AGEs. Data quantification of 72kDa bands is shown in a', b', c', d' and e', respectively. The density of the saline control was set as 1.0. All values are expressed as mean±S.E.M. The P value is obtained from comparative analysis of indicated group with the control.

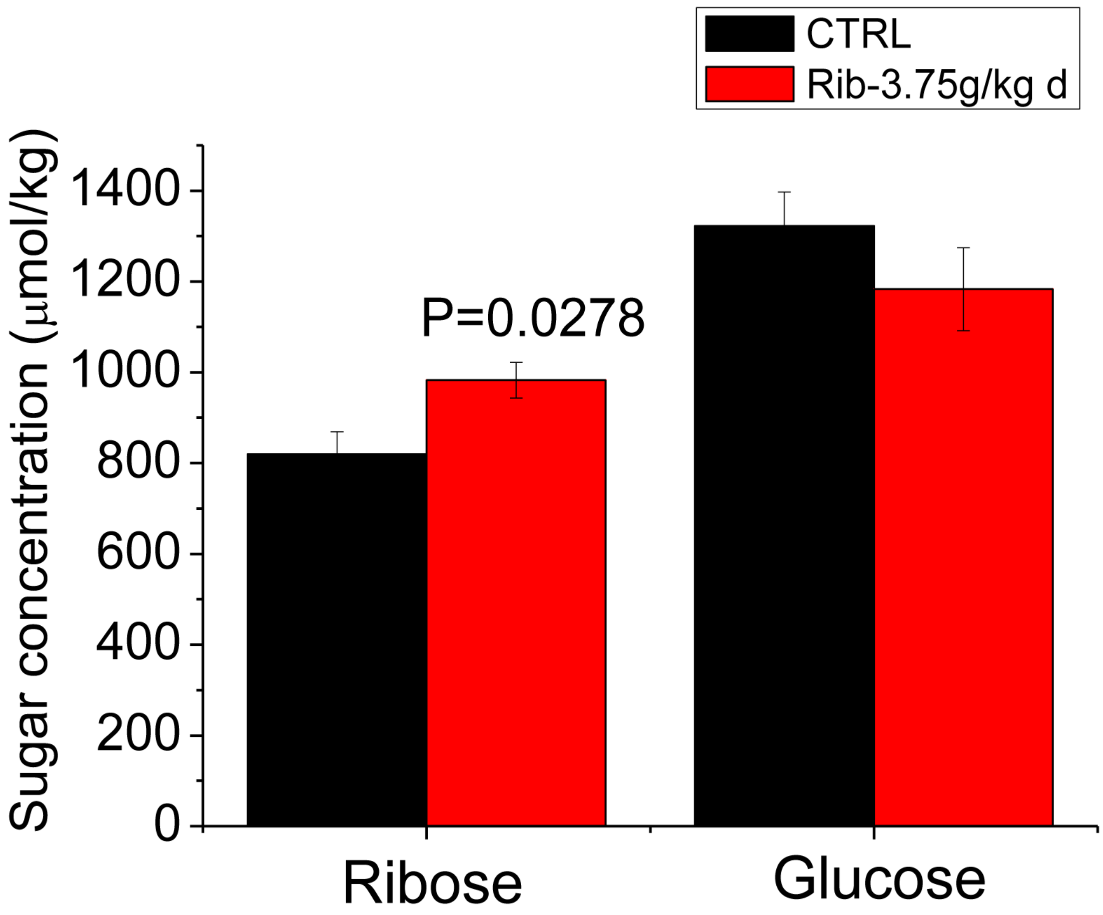


**Supplementary Figure 13: Changes in levels of D-Ribose and D-Glucose in the brains of sugar-treated mice.** Conditions for the treatment were the same as those given in Figure 1. The brain D-Ribose and D-Glucose concentrations were measured by UV-HPLC. The P value is obtained from comparative analysis of indicated group with the control.

Hippocampus

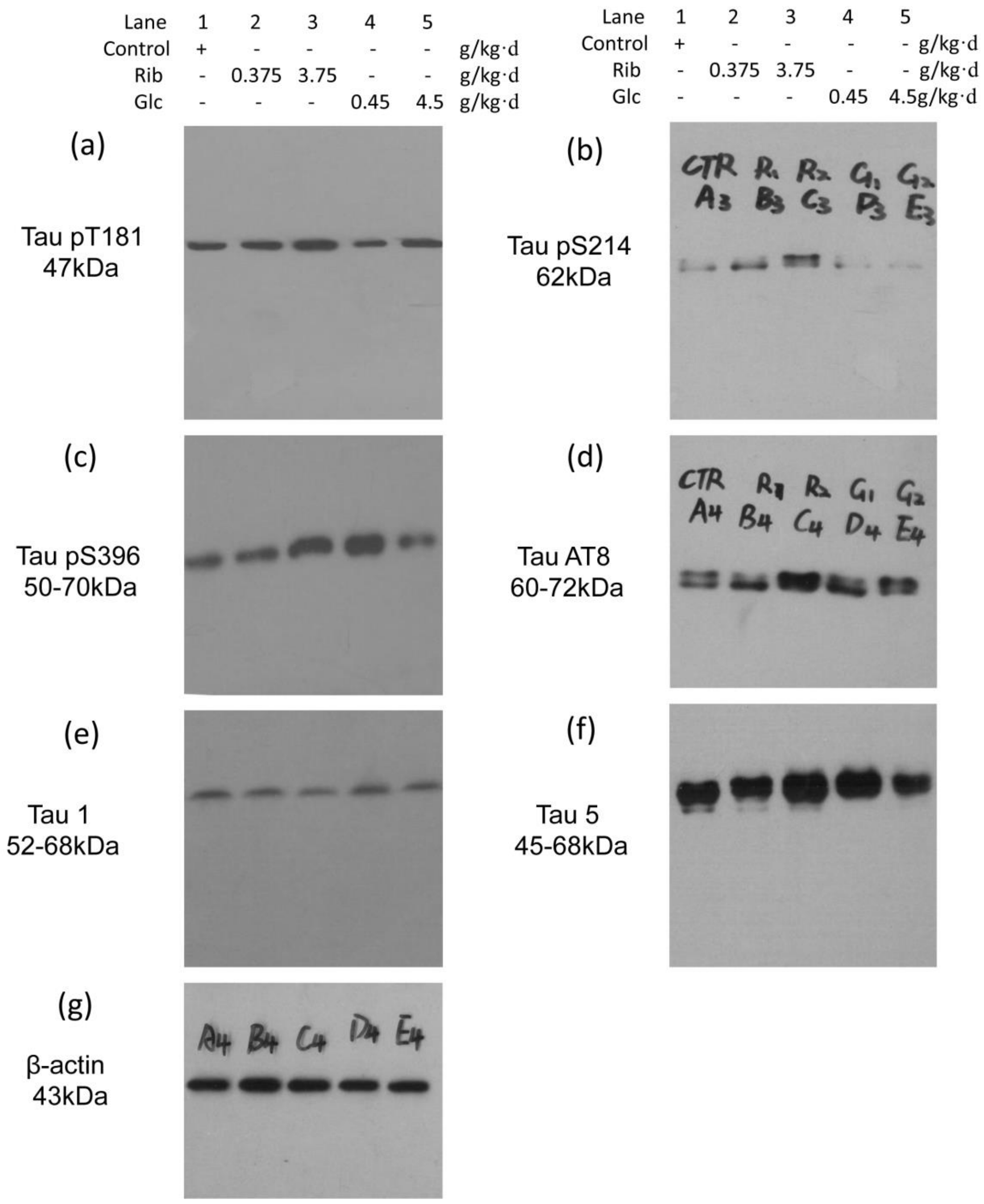

Cortex

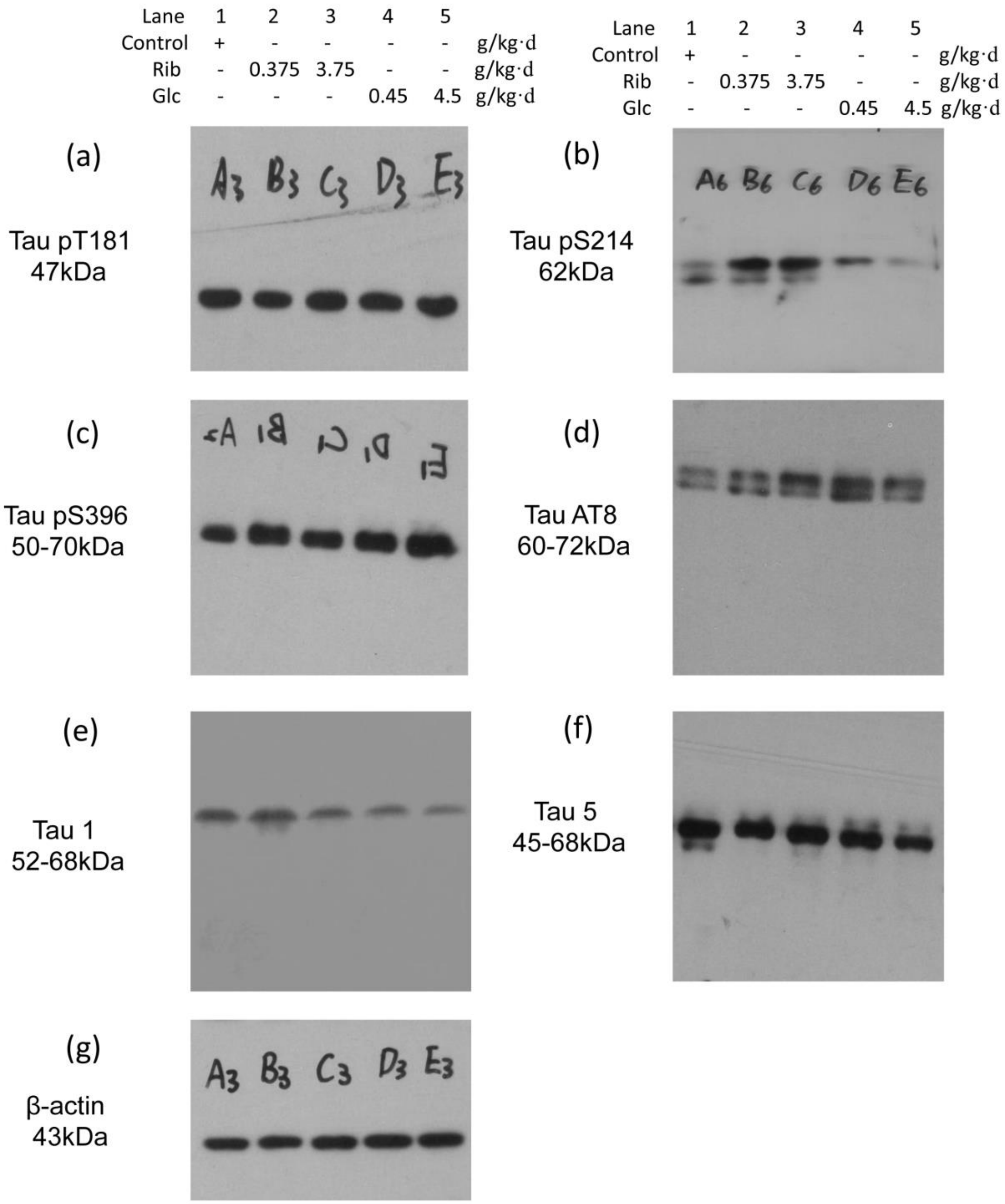


**Supplementary Figure 14 and 15: Showed the full-length blots of Figure 3.**

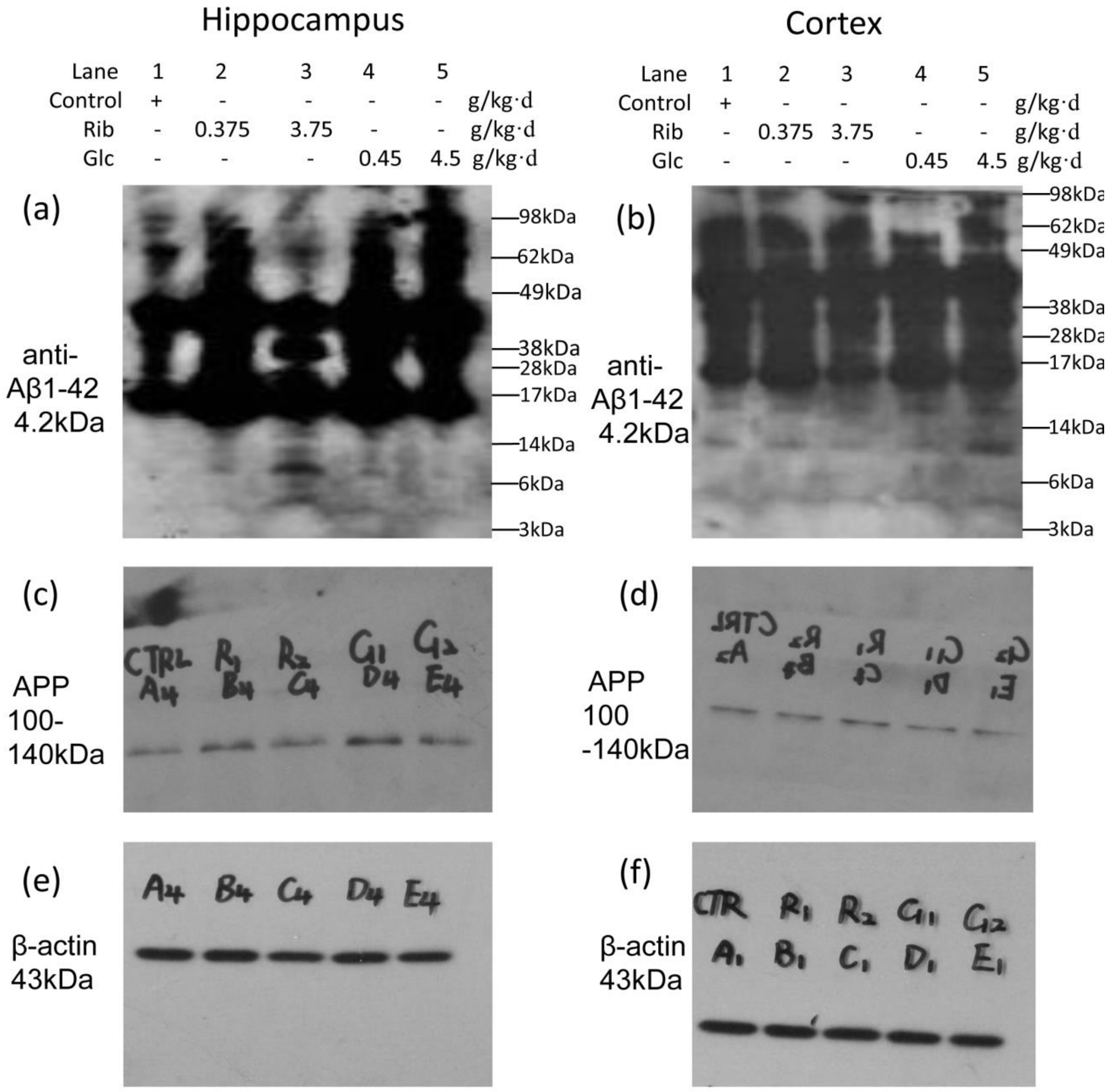


**Supplementary Figure 16: Showed the full-length blots of Figure 5.**
